# Ultra-processed foods, adiposity and risk of head and neck cancer and oesophageal adenocarcinoma in the European Prospective Investigation into Cancer and Nutrition study: a mediation analysis

**DOI:** 10.1007/s00394-023-03270-1

**Published:** 2023-11-22

**Authors:** Fernanda Morales‑Berstein, Carine Biessy, Vivian Viallon, Ana Goncalves‑Soares, Corinne Casagrande, Bertrand Hémon, Nathalie Kliemann, Manon Cairat, Jessica Blanco Lopez, Aline Al Nahas, Kiara Chang, Eszter Vamos, Fernanda Rauber, Renata Bertazzi Levy, Diana Barbosa Cunha, Paula Jakszyn, Pietro Ferrari, Paolo Vineis, Giovanna Masala, Alberto Catalano, Emily Sonestedt, Yan Borné, Verena Katzke, Rashmita Bajracharya, Claudia Agnoli, Marcela Guevara, Alicia Heath, Loredana Radoï, Francesca Mancini, Elisabete Weiderpass, José María Huerta, María‑José Sánchez, Anne Tjønneland, Cecilie Kyrø, Matthias B. Schulze, Guri Skeie, Marko Lukic, Tonje Braaten, Marc Gunter, Christopher Millett, Antonio Agudo, Paul Brennan, M. Carolina Borges, Rebecca C. Richmond, Tom G. Richardson, George Davey Smith, Caroline L. Relton, Inge Huybrechts

**Affiliations:** 1 MRC Integrative Epidemiology Unit, University of Bristol, Bristol, UK; 2 Population Health Sciences, Bristol Medical School, University of Bristol, Bristol, UK; 3 Nutrition and Metabolism Branch, International Agency for Research on Cancer/World Health Organization, Lyon, France; 4 Cancer Research Center of Santa Catarina, CEPON, Florianópolis, Brazil; 5 Paris-Saclay University, UVSQ, Inserm “Exposome, Heredity, Cancer and Health” Team, CESP U1018, Gustave Roussy, Villejuif, France; 6 Public Health Policy Evaluation Unit, School of Public Health, Imperial College London, London, UK; 7 Preventive Medicine Department of the Medical School, University of São Paulo, São Paulo, Brazil; 8 Center for Epidemiological Research in Nutrition and Health, University of São Paulo, São Paulo, Brazil; 9 Hésio Cordeiro Institute of Social Medicine, Department of Epidemiology, Rio de Janeiro State University, Rio de Janeiro, RJ, Brazil; 10 Unit of Nutrition and Cancer, Catalan Institute of Oncology-ICO, L’Hospitalet de Llobregat, Spain; 11 Nutrition and Cancer Group; Epidemiology, Public Health, Cancer Prevention and Palliative Care Program, Bellvitge Biomedical Research Institute-IDIBELL, L’Hospitalet de Llobregat, Spain; 12 Blanquerna Faculty of Health Sciences, Ramon Llull University, Barcelona, Spain; 13 MRC Centre for Environment and Health, School of Public Health, Imperial College London, Norfolk Place, London W2 1PG, UK; 14 Italian Institute of Technology, Genoa, Italy; 15 Institute for Cancer Research, Prevention and Clinical Network (ISPRO), Florence, Italy; 16 Centre for Biostatistics, Epidemiology, and Public Health, Department of Clinical and Biological Sciences, University of Turin, 10043 Orbassano, TO, Italy; 17 Nutrition Epidemiology, Department of Clinical Sciences Malmö, Faculty of Medicine, Lund University, Lund, Sweden; 18 Department of Cancer Epidemiology, German Cancer Research Center (DKFZ), Heidelberg, Germany; 19 Epidemiology and Prevention Unit, Fondazione IRCCS Istituto Nazionale dei Tumori, Milan, Italy; 20 Instituto de Salud Pública y Laboral de Navarra, 31003 Pamplona, Spain; 21 Centro de Investigación Biomédica en Red de Epidemiología y Salud Pública (CIBERESP), 28029 Madrid, Spain; 22 Navarra Institute for Health Research (IdiSNA), 31008 Pamplona, Spain; 23 Department of Epidemiology and Biostatistics, School of Public Health, Imperial College London, London, UK; 24 International Agency for Research on Cancer, World Health Organization, Lyon, France; 25 Department of Epidemiology, Murcia Regional Health Council-IMIB, Murcia, Spain; 26 Escuela Andaluza de Salud Pública (EASP), 18011 Granada, Spain; 27 Instituto de Investigación Biosanitaria ibs.GRANADA, 18012 Granada, Spain; 28 Department of Preventive Medicine and Public Health, University of Granada, 18071 Granada, Spain; 29 Danish Cancer Society Research Center, Diet, Cancer and Health, Strandboulevarden 49, DK-2100 Copenhagen, Denmark; 30 Department of Public Health, University of Copenhagen, DK-2200 Copenhagen, Denmark; 31 Department of Molecular Epidemiology, German Institute of Human Nutrition Potsdam-Rehbruecke, Nuthetal, Germany; 32 Institute of Nutritional Science, University of Potsdam, Nuthetal, Germany; 33 Department of Community Medicine, Faculty of Health Sciences, UiT The Arctic University of Norway, Tromsø, Norway; 34 NOVA National School of Public Health, Public Health Research Centre, Comprehensive Health Research Center, CHRC, NOVA University Lisbon, Lisbon, Portugal; 35 Genetic Epidemiology Group, International Agency for Research on Cancer, World Health Organization, Lyon, France

**Keywords:** Epidemiology, Food processing, NOVA classification, Oesophageal cancer, Head and neck cancer, Adiposity, Mediation analysis

## Abstract

**Purpose:**

To investigate the role of adiposity in the associations between ultra-processed food (UPF) consumption and head and neck cancer (HNC) and oesophageal adenocarcinoma (OAC) in the European Prospective Investigation into Cancer and Nutrition (EPIC) cohort.

**Methods:**

Our study included 450,111 EPIC participants. We used Cox regressions to investigate the associations between the consumption of UPFs and HNC and OAC risk. A mediation analysis was performed to assess the role of body mass index (BMI) and waist-to-hip ratio (WHR) in these associations. In sensitivity analyses, we investigated accidental death as a negative control outcome.

**Results:**

During a mean follow-up of 14.13 ± 3.98 years, 910 and 215 participants developed HNC and OAC, respectively. A 10% g/d higher consumption of UPFs was associated with an increased risk of HNC (hazard ratio [HR] = 1.23, 95% confidence interval [CI] 1.14–1.34) and OAC (HR = 1.24, 95% CI 1.05–1.47). WHR mediated 5% (95% CI 3–10%) of the association between the consumption of UPFs and HNC risk, while BMI and WHR, respectively, mediated 13% (95% CI 6–53%) and 15% (95% CI 8–72%) of the association between the consumption of UPFs and OAC risk. UPF consumption was positively associated with accidental death in the negative control analysis.

**Conclusions:**

We reaffirmed that higher UPF consumption is associated with greater risk of HNC and OAC in EPIC. The proportion mediated via adiposity was small. Further research is required to investigate other mechanisms that may be at play (if there is indeed any causal effect of UPF consumption on these cancers).

## Introduction

In recent years, there has been growing interest in the potential role of industrial food processing in disease aetiology. The NOVA (not an abbreviation) classification system developed by Monteiro et al. [1, 2] categorises foods into four groups according to their degree and purpose of processing: (1) unprocessed or minimally processed foods, (2) processed culinary ingredients, (3) processed foods and (4) ultra-processed foods (UPFs). UPFs are industrial formulations manufactured in a complex way using ingredients not usually found in kitchens (e.g. maltodextrin, hydrogenated oils, modified starches) and cosmetic additives (e.g. emulsifiers, flavourings, colourants, artificial sweeteners) [2]. They are typically cheap, highly palatable, and widely available ready-to-eat products which are often consumed in large quantities, replacing more nutritious, unprocessed/minimally processed foods in the diet [3, 4]. Examples of UPFs include soft drinks, sweet or savoury packaged snacks, confectionery, packaged breads and buns, reconstituted meat products and pre-prepared frozen or shelf-stable dishes.

Several studies have shown that the consumption of UPFs may be associated with an increased risk of cancer [5–9]. In the European Prospective Investigation into Cancer and Nutrition (EPIC) cohort, Kliemann et al. [9] found positive associations between higher UPF consumption and the risk of head and neck cancer (HNC; hazard ratio [HR] = 1.14 per one standard deviation [SD] higher UPF intake, 95% confidence interval [CI] 1.06–1.24) and oesophageal adenocarcinoma (OAC; HR = 1.21 per 1-SD higher UPF intake, 95% CI 1.05–1.39). They also found an inverse association between UPF consumption and oesophageal squamous cell carcinoma risk (HR = 0.79 per 1-SD higher UPF intake, 95% CI 0.64–0.96), although this did not withstand additional adjustments for alcohol intake, body mass index (BMI) and several dietary factors (HR = 0.90 per 1-SD higher UPF intake, 95% CI 0.72–1.11).

UPF consumption has also been positively associated with higher adiposity (i.e. BMI, fat mass, waist circumference and waist-to-hip ratio [WHR]) [10–14]. Since body fatness (measured by BMI, waist circumference and WHR) is an established modifiable risk factor for OAC [15–20], and visceral adiposity (i.e. waist circumference and WHR) has been positively associated with HNC risk [21–23], it is plausible that the positive associations between UPF consumption and these upper-aerodigestive tract cancers are mediated via adiposity. Although BMI has been inversely associated with HNC risk, this seems to be a consequence of residual confounding related to smoking (an established risk factor for HNC), as smokers tend to have lower BMIs than non-smokers [23]. In a meta-analysis of 20 prospective cohort studies, BMI was positively associated with HNC risk when the analysis was restricted to never smokers [22]. Although adiposity may be one of the mechanisms underlying the association between UPF consumption and upper-aerodigestive tract cancer, this has not been investigated using mediation analysis.

The aim of this study was to reassess and further investigate the associations between the consumption of UPFs and the risk of HNC and OAC in the EPIC study. As a complement to the study by Kliemann et al. [9] (described above), this study explored the associations between UPF consumption and the risk of HNC and its subtypes (i.e. oral cavity, oropharynx, hypopharynx, larynx and unspecified/overlapping cancers) as defined by the International Head and Neck Cancer Epidemiology (INHANCE) consortium. It also investigated effect modification by smoking status, alcohol intake, sex, physical activity, and education level in the associations between the consumption of UPFs and the risk of upper-aerodigestive tract cancers. Additionally, this study assessed the possibility of residual confounding using accidental death as a negative control outcome. Lastly, it examined the role of BMI and WHR in the associations between UPF consumption and the risk of HNC and OAC by means of a mediation analysis.

## Methods

### The EPIC cohort

The EPIC study has been fully described elsewhere [24–26]. Briefly, EPIC is one of the largest prospective cohort studies in Europe. It recruited 521,323 participants between 1992 and 2000. Participants were enrolled in 23 centres across 10 European countries, namely Denmark, France, Germany, Greece, Italy, the Netherlands, Norway, Spain, Sweden and the United Kingdom. Most were 35–69 years old at recruitment [24, 25]. They were either volunteers from the general population, blood donors, employees of local companies, teachers/school employees or individuals enrolled in local ongoing studies. All participants provided written informed consent before completing the dietary and lifestyle questionnaires. Anthropometric and blood pressure data were also obtained at baseline. EPIC was approved by the International Agency for Research on Cancer (IARC) Ethics Committee and the local ethical review boards of all EPIC centres.

### Study sample

Participants who withdrew consent from the study were not included in this research. We excluded participants diagnosed with cancer before enrolment (*n* = 25,184) and those with a length of follow-up equal to zero (*n* = 4148). We also excluded participants who did not complete the dietary or lifestyle questionnaires (*n* = 6259). We additionally excluded participants with extreme energy intake versus energy requirement ratios (top and bottom 1%) (*n* = 9573) and participants recruited in Greece due to administrative issues (*n* = 26,048). After exclusions, 450,111 participants were included in the analyses (Supplementary Fig. 1).

### Dietary data and food processing variables

Semi-quantitative food frequency questionnaires (FFQs), extensive quantitative dietary questionnaires, and combined methods (i.e. semi-quantitative FFQs combined with 7-day records in the UK, and a non-quantitative FFQ combined with a 14-day record on hot meals in Malmö, Sweden) were used to obtain dietary data at baseline [25]. These were centre specific to account for local dietary habits and were either self-administered or administered in-person by trained interviewers. Furthermore, a standardised 24-h recall was used to obtain supplementary dietary data for a subsample of EPIC participants to calibrate baseline dietary measurements across EPIC centres [25, 27–30]. The dietary questionnaires and their mode of administration were described in detail in previous publications [25, 30].

The NOVA classification was used to categorise foods into four groups according to their extent and purpose of industrial processing [31]. Unprocessed/minimally processed foods (NOVA 1) are natural foods that may have undergone minimal processing for their preservation, storage, safety, or edibility. Processed culinary ingredients (NOVA 2) correspond to substances derived from unprocessed/minimally processed foods (e.g. oil, butter) or nature (e.g. salt) that are normally consumed in combination with unprocessed/minimally processed foods. Both processed foods (NOVA 3) and UPFs (NOVA 4) are industrial products. The former typically contain two or three common ingredients (i.e. a combination of unprocessed/minimally processed foods and processed culinary ingredients), while the latter contain many ingredients (most of which are rarely used in kitchens) and additives that make the final product tastier and more attractive to consumers.

Food preparations made (at home or elsewhere) using traditional methods were decomposed using standardised recipes. Individual food items were then classified according to their degree of processing. Food items were combined into broader food categories for simplicity. Of a total of 67 food categories in the dietary questionnaires, 19 were classified as unprocessed/minimally processed foods, 5 as culinary ingredients, 13 as processed foods and 30 as UPFs (see Supplementary Table 1 for details).

Here, we used the relative intake of each NOVA group in grams per day (%g/d). We also used the absolute intake in grams per day (g/d) and the absolute and relative intake in kilocalories per day in sensitivity analyses (kcal/d and %kcal/d, respectively).

### Ascertainment of cancer cases

Incident cancer cases were identified through population-based cancer registries in Denmark, Italy (except Naples), the Netherlands, Norway, Spain, Sweden and the United Kingdom. Participants in other centres (France, Germany, Greece and Naples) were actively followed up using health insurance records, pathology registries and direct contact with participants or their next of kin.

HNC and OAC were defined using the 2nd and 3rd Revision of the International Classification of Diseases for Oncology (ICDO-2 and ICDO-3). According to the INHANCE consortium [32], HNC cases include malignant neoplasms of the oral cavity (topography codes C00.3–C00.6, C00.8–C00.9, C02.0–C02.3, C03.0–C03.1, C03.9, C04.0–C04.1, C04.8–C04.9, C05.0, C06.0–C06.2, C06.8–C06.9), oropharynx (C01.9, C02.4, C05.1–C05.2, C09.0–C09.1, C09.8–C09.9, C10.0–C10.4, C10.8–C10.9), hypopharynx (C12.9–C13.2, C13.8–C13.9), larynx (C32.0–C32.3, C32.8–C32.9), and oral cavity and pharynx unspecified/overlapping regions (C02.8–C02.9, C05.8–C05.9, C14.0, C14.2, C14.8). We did not exclude any histological subtypes of HNC. Oesophageal cancer cases correspond to topography codes C15.0–C15.5 and C15.8–C15.9. Among these, OAC cases were identified with codes 8140/3, 8144/3, 8480/3, 8481/3 and 8490/3. Other oesophageal cancer subtypes (e.g. squamous cell carcinoma and small cell carcinoma) were not investigated as outcomes in this study.

### Covariates

Data on age at recruitment, sub-centre (22 centres in total, split into 27 sub-centres as follows: Northeast of France, Northwest of France, South of France, South coast of France, Florence, Varese, Ragusa, Turin, Naples, Asturias, Granada, Murcia, Navarra, San Sebastian, Cambridge, Oxford health-conscious population, Oxford general population, Bilthoven, Utrecht, Heidelberg, Potsdam, Malmö, Umeå, Aarhus, Copenhagen, Southeast of Norway, Northwest of Norway), sex (male/female), education level (none, primary, technical/professional, secondary, further education), physical activity based on the Cambridge Physical Activity Index [33] (inactive, moderately inactive, moderately active, active), measured/self-reported height (continuous in cm) and smoking status (never, former, current, unknown) were obtained at baseline through anthropometric measurements and lifestyle questionnaires. Additionally, data on alcohol intake (continuous in g/d) were acquired using dietary questionnaires.

### Potential mediators

BMI and WHR were investigated as potential mediators in mediation analyses. BMI (continuous in kg/m^2^) was calculated from measured height and weight (measured using comparable, standardised methods) [34]. WHR (continuous) was estimated from measured waist and hip circumferences. Waist circumference was measured midway between the iliac crest and the lower ribs or at the narrowest torso circumference. Hip circumference was measured over the buttocks or at the widest point. EPIC-Oxford health-conscious population self-reported data were also used to estimate BMI and WHR, after the application of measurement error corrections [34, 35].

### Statistical analysis

#### Descriptive characteristics

The participants’ baseline characteristics were divided into sex-specific quartiles of relative UPF consumption (in %g/d). Mean and SD estimates were obtained for continuous variables, while frequencies and percentages were obtained for binary/categorical variables. Furthermore, we made a histogram to graphically represent the distribution of UPF consumption (in %g/d) in the EPIC cohort.

#### Data imputation

We used single-value imputation to deal with missing data in the covariates used to control for potential confounding (i.e. height, physical activity, education level and smoking status). When measured/self-reported height values were not available, missing values were imputed with mean centre-, age- and sex-specific height values [34]. Mode imputation was used for baseline binary and categorical covariates missing less than 5% of their values (i.e. education level: “primary school completed”, physical activity: “moderately inactive”, smoking status: “never”). Multiple imputation was used in sensitivity analyses (details in the “sensitivity analyses” subsection below).

#### Main association analysis

Cox proportional hazards models with age as the underlying timescale were used to investigate the association between the intake of UPFs and the risk of HNC and OAC. We estimated HRs and 95% CIs per 10% g/d higher consumption of UPFs. Time of entry was defined as age at recruitment, while time of exit was defined as age at first cancer diagnosis (excluding non-melanoma skin cancer) or age at last follow-up (i.e. death, emigration, loss to follow-up or end of follow-up [i.e. between June 2008 and December 2013, depending on the centre]), whichever came first. Model 1 was stratified by age at recruitment in 1-year categories, sex and sub-centre. Model 2 was additionally adjusted for education, physical activity, height and smoking status. Model 3 was additionally adjusted for alcohol intake in g/d to reflect the association between the consumption of UPFs and cancer, regardless of alcohol intake (a well-known cancer risk factor [36–44] that forms part of some processed foods and UPFs).

We graphically assessed the proportional hazards assumption using log–log survival plots. Additionally, we tested proportionality using Schoenfeld residuals. We also used correlation matrices and variance inflation factors to assess the presence of multicollinearity. Non-linearity was assessed using likelihood ratio tests comparing UPF consumption (in %g/d) modelled with and without natural cubic splines.

We undertook additional analyses to investigate the associations between the consumption of UPFs and the risk of HNC subtypes (i.e. oral cavity, oropharynx, hypopharynx, larynx, and oral cavity and pharynx unspecified/overlapping cancers). Heterogeneity tests were used to assess differences between HNC subtype estimates.

Furthermore, we stratified Model 3 (for every exposure–outcome combination) by alcohol intake (as defined by Wozniak et al. [45], i.e. no/light alcohol intake [0.1–6 g/d (men); 0.1–3 g/d (women)], moderate alcohol intake [6.1–24 g/d (men); 3.1–24 g/d (women)], heavy alcohol intake [> 24 g/d]), sex (i.e. male, female), physical activity (i.e. inactive, moderately inactive, moderately active, active), smoking status (i.e. never smoker, former smoker, current smoker) and education level (i.e. primary school or less, secondary or technical/professional school, higher education) and performed likelihood ratio tests to explore interactions. Models were not adjusted for the stratification variable.

#### Mediation analysis

**Fig. 1:**
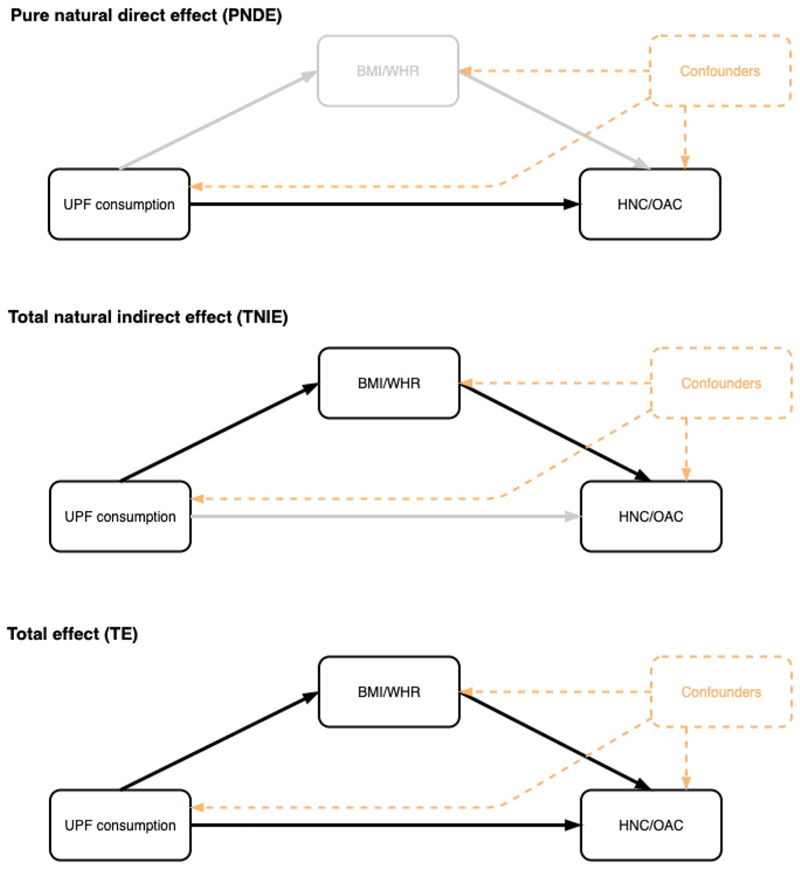
Mediation analysis diagram of the counterfactual two-way decomposition of the total effect of UPF consumption on the risk of head and neck cancer and oesophageal adenocarcinoma. All mediation models accounted for potential exposure–mediator interactions and were adjusted for age at recruitment in 1-year categories, sex, sub-centre, education, physical activity, height, smoking status and alcohol intake. The total effect (TE) corresponds to the sum of the pure natural direct effect (PNDE) and the total natural indirect effect (TNIE). Point estimates were obtained by direct counterfactual imputation estimation and confidence intervals were obtained using 1000 bootstrap repetitions. Abbreviations: BMI, body mass index; WHR, waist-to-hip ratio; UPF, ultra-processed food; HNC, head and neck cancer, OAC, oesophageal adenocarcinoma

Under the strong assumption that there is no residual confounding or measurement error in our study, we conducted a mediation analysis using the counterfactual framework [46] to further explore the mediating role of BMI and WHR in the associations between UPF consumption and the risk of HNC and OAC (Fig. 1).

In exploratory analyses, we ran linear regressions to study the associations between UPF consumption (i.e. the exposure) and both WHR and BMI (i.e. the potential mediators). We also ran exposure-adjusted Cox regressions to analyse the associations between the potential mediators and the risk of both HNC and OAC (i.e. the outcomes). Where there was evidence of an association between the potential mediator and both the exposure and the outcome, we used the “cmest” function in the “CMAverse” R package [47] to decompose the Total Effect (TE) of UPF consumption on the corresponding upper-aerodigestive tract cancer into a Pure Natural Direct Effect (PNDE) and a Total Natural Indirect Effect (TNIE) (on the ratio scale TE = PNDE × TNIE). The proportion mediated was also calculated (i.e. 100 × (PNDE × (TNIE – 1))/(TE – 1)) for each exposure–mediator–outcome combination [48]. All mediation models accounted for potential exposure–mediator interactions and were adjusted for age at recruitment in 1-year categories, sex, sub-centre, education, physical activity, height, smoking status and alcohol intake. Point estimates were obtained by direct counterfactual imputation estimation and 95% CIs were obtained using 1000 bootstrap repetitions. The results were scaled to reflect a 10% g/d higher consumption of UPFs.

#### Sensitivity analyses

As a sensitivity analysis, we explored adjusting our Cox models for total water intake (i.e. water content from foods and drinks, in addition to drinking water and water used as an ingredient in preparations). This was to see whether differences in water content across NOVA groups may influence the associations between the relative intake of UPFs and the risk of HNC and OAC. Similarly, we explored adjustments for total energy intake.

We also reran our Cox models after excluding participants who were censored during the first two years of follow-up to avoid reverse causation due to undiagnosed cancer at recruitment.

Additionally, we repeated the analyses using the absolute intake of UPFs in grams per day (g/d) and the absolute and relative intake in kilocalories per day (kcal/d and %kcal/d, respectively) as the exposure.

Moreover, we conducted a complete case analysis excluding participants with missing data for at least one lifestyle covariate (i.e. smoking status, physical activity and education level). In addition, we used the ‘mice’ R package to perform multivariate imputation by chained equations (MICE) [49], whereby smoking status, physical activity and education level were imputed five times by predictive mean matching. We fit our models using the MICE imputed data sets and then pooled the results according to Rubin’s rules [50] to obtain average HR estimates and standard errors for each model. For the complete case analysis and the MICE analysis, we still used centre-, age- and sex-specific imputed height as a covariate, as this is standard practice when dealing with anthropometric variables as confounders in EPIC [34].

Finally, we performed a negative control outcome analysis (i.e. where the outcome is not plausibly linked to the exposure of interest) to help identify any residual confounding that could be biasing our results [51]. We considered accidental deaths as the outcome (instead of upper-aerodigestive tract cancers) since the consumption of foods by their degree of processing is unlikely associated with the risk of being involved in a deadly accident (e.g. falls, transport accidents, accidental drowning). Any evidence of an association between UPF consumption and accidental deaths would suggest that our main results may be biased by the same factors that biased the negative control outcome results. Accidental deaths were defined as deaths due to events linked to codes V01–X59 in the 10th Revision of the International Classification of Diseases (ICD-10). For the negative control analysis, time of exit was defined as age at the time of death, emigration, loss to follow-up or end of follow-up, whichever came first. Participants were not censored at the time of cancer diagnosis, whereas they were in all other analyses in this study. The accidental death models accounted for the same covariates as the main analysis. BMI and type 2 diabetes mellitus would not normally be adjusted for in this analysis, as they are potential mediators and adjusting for them could induce collider bias (i.e. open backdoor paths from UPF consumption to accidental deaths through unobserved factors) [52]. Here, we did this in an explorative manner, assuming the absence of unobserved confounders of BMI, type 2 diabetes mellitus and accidental deaths.

#### Statistical software

All statistical analyses and visualisations were performed using R version 4.2.3. We used version 3.2.10 of the “survival” R package for the Cox regressions and version 0.1.0 of the “CMAverse” R package [47] for the mediation analysis. We also used version 0.1.0 of the “ggforestplot” R package to create forest plots. To create tables, we used version 1.3.0 of the “tidyverse” R package and version 0.7.0 of the “flextable” R package. P-values for heterogeneity between HNC subtype estimates were obtained using version 4.18–0 of the “meta” R package. Non-linearity was assessed using version 4.2.3 of the “splines” R package. MICE was performed using version 3.16 of the “mice” R package. Two-sided *p*-values < 0.05 were considered statistically significant.

## Results

### Descriptive characteristics

In total, we included 450,111 participants of which 70.8% were female. The mean age at recruitment was 51.1 years (SD 9.8, range 17.8–98.5 years). The mean consumption of UPFs in the cohort was 13.7% g/d (364 g/d), ranging from a mean intake of 8% g/d (156.9 g/d) in Spain to 18.6% g/d (520.5 g/d) in the United Kingdom. A histogram of the proportion of UPFs in the diet (in %g/d) is available in Supplementary Fig. 2. On average, males consumed a higher proportion of UPFs than females (14.7% vs 13.3%, *p* < 0.001). Participants with technical education were among the highest consumers of UPFs (Table 1). UPFs contributed greatly to the diet of younger, taller, and more physically active participants. Participants who did not provide data on their physical activity and education also tended to consume more UPFs. In terms of diet quality, UPFs were highly consumed by participants who consumed less alcohol and more calories, carbohydrates, fat and sodium.

The UPF group was mainly composed of fizzy drinks (14.1% of absolute UPF consumption in g/d), non-carbonated sweetened beverages (12.1%), ultra-processed dairy products (12.0%), ultra-processed breads (12.0%) and ultra-processed meats (9.9%) (Supplementary Table 1). Beer and wine (46.2%) were the main contributors to the processed foods group, followed by processed breads (22.7%) and cheese (10.2%). The unprocessed/minimally processed foods group primarily comprised tea and coffee (32.2%), water (17.3%), milk and plain yoghurt (12.4%), fruit (11.2%) and vegetables (8.9%).

### Associations between the consumption of ultra-processed foods and upper-aerodigestive tract cancers

During a mean follow-up of 14.13 ± 3.98 years (6,358,569 person-years, median follow-up = 14.95 years; range 1 day–22.79 years), 1125 incident cases of HNC and OAC were documented. Of these, 910 had HNC (i.e. 234 oral cavity, 235 oropharynx, 66 hypopharynx, 310 larynx and 65 oral cavity and pharynx unspecified/overlapping regions) and 215 had OAC.

The proportional hazards assumption was met in all models (Supplementary Figs. 3, 4, 5, 6), and we did not find evidence of multicollinearity between covariates (Supplementary Fig. 7 and Supplementary Table 2). Furthermore, there was no evidence of non-linearity between UPF consumption and HNC (*p* = 0.54) (Supplementary Fig. 8). The non-linearity test for the association between UPF consumption and the risk of OAC was not informative due to the limited number of OAC cases in the dataset (results not shown).

#### Head and neck cancer (HNC)

**Fig. 2:**
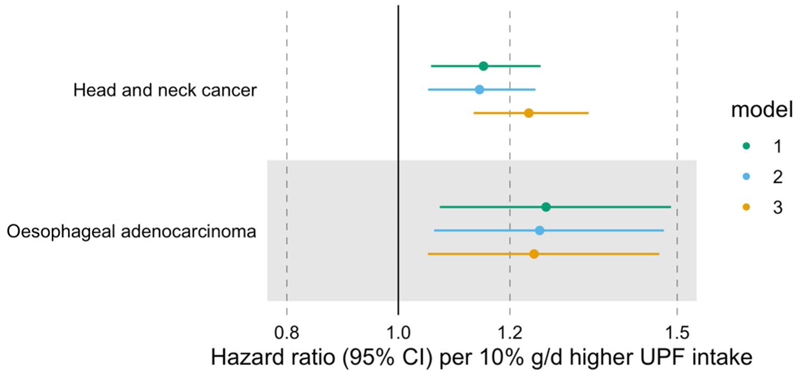
Associations between the consumption of ultra-processed foods (in %g/d) and the risk of head and neck cancer and oesophageal adenocarcinoma. Hazard ratios per 10% g/d higher ultra-processed food intake. Time of entry was defined as age at recruitment, while time of exit was defined as age at first cancer diagnosis (excluding non-melanoma skin cancer) or age at last follow-up (i.e. death, emigration, loss to follow-up or end of follow-up), whichever came first. Model 1 was stratified by age at recruitment in 1-year categories, sex, and sub-centre. Model 2 was additionally adjusted for education, physical activity, height, and smoking status. Model 3 was additionally adjusted for alcohol intake. *N* = 450,111, of which 910 and 215 had head and neck cancer and oesophageal adenocarcinoma, respectively. Abbreviations: CI, confidence interval; UPF, ultra-processed food

**Fig. 3:**
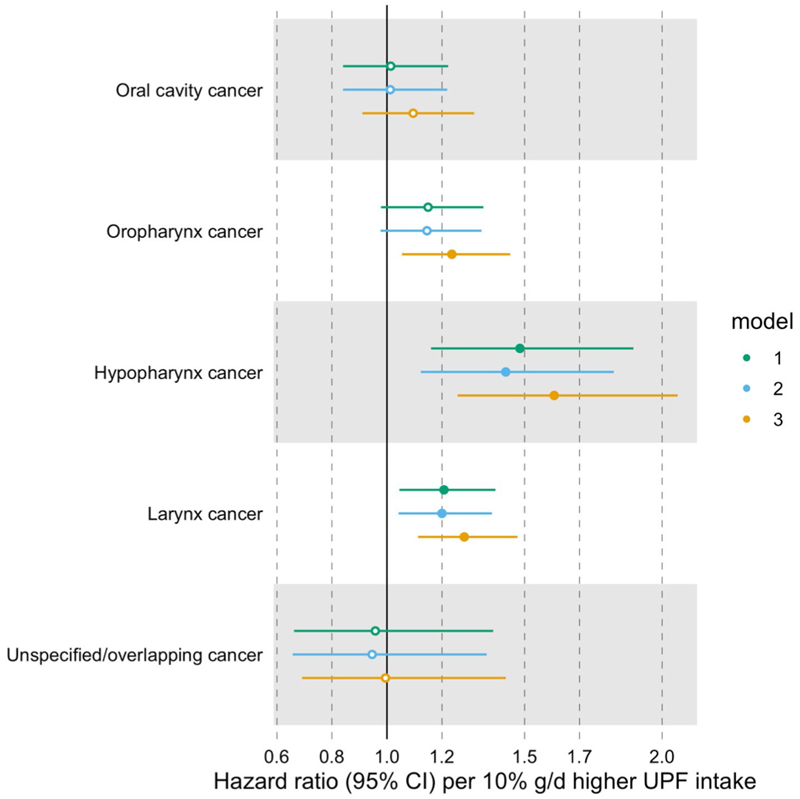
Associations between the consumption of ultra-processed foods (in %g/d) and head and neck cancer subtypes. Hazard ratios per 10% g/d higher ultra-processed food intake. Time of entry was defined as age at recruitment, while time of exit was defined as age at first cancer diagnosis (excluding non-melanoma skin cancer) or age at last follow-up (i.e. death, emigration, loss to follow-up or end of follow-up), whichever came first. Model 1 was stratified by age at recruitment in 1-year categories, sex, and sub-centre. Model 2 was additionally adjusted for education, physical activity, height and smoking status. Model 3 was additionally adjusted for alcohol intake. *N* = 450,111, of which 234, 235, 66, 310 and 65 had cancer of the oral cavity, oropharynx, hypopharynx, larynx, and oral cavity and pharynx unspecified/overlapping regions, respectively. Abbreviations: CI, confidence interval; UPF, ultra-processed food

A higher proportion of UPF in the diet (in %g/d) was associated with a higher risk of HNC, even after accounting for alcohol intake (HR = 1.23 per 10% g/d higher UPF intake, 95% CI 1.14–1.34) (Fig. 2 and Supplementary Table 3). We did not find evidence of heterogeneity between HNC subtypes (*p*-value for heterogeneity = 0.11) (Fig. 3 and Supplementary Table 4).

In stratified analyses for the association between UPF consumption (in %g/d) and HNC risk, we did not find evidence of effect modification by alcohol intake (*p*-value for interaction = 0.46), physical activity level (*p*-value for interaction = 0.48), smoking status (*p*-value for interaction = 0.46) or education level (*p*-value for interaction = 0.31) (Supplementary Table 5). There was some evidence of an interaction by sex (*p*-value for interaction = 0.006), with a positive association between UPF consumption and HNC risk among males (HR = 1.34 per 10% g/d higher UPF intake, 95% CI 1.22–1.48, *N* = 131,425, events = 603) but not among females (HR = 1.03, 95% CI 0.87–1.21, *N* = 318,686, events = 307).

#### Oesophageal adenocarcinoma (OAC)

After accounting for alcohol intake, UPF consumption (in %g/d) was positively associated with OAC risk (HR = 1.24 per 10% g/d higher UPF intake, 95% CI 1.05–1.47) (Fig. 2 and Supplementary Table 3).

When we stratified the association between UPF consumption (in %g/d) and OAC risk, we did not find evidence of differing estimates across levels of alcohol intake (*p*-value for interaction = 0.18), physical activity (*p*-value for interaction = 0.94), smoking status (*p*-value for interaction = 0.99), sex (*p*-value for interaction = 0.44) or education (*p*-value for interaction = 0.83) (Supplementary Table 5).

### Mediating role of adiposity in the associations between ultra-processed food consumption and upper-aerodigestive tract cancers

In exploratory analyses (Supplementary Table 6), we found positive associations between UPF consumption and both BMI (mean change = 0.24 kg/m^2^ per 10% g/d higher UPF intake, 95% CI 0.22–0.26) and WHR (mean change = 0.41 per 10% g/d higher UPF intake, 95% CI 0.38–0.43). Moreover, BMI was positively associated with OAC risk (HR = 1.08 per 1 kg/m^2^ higher BMI, 95% CI 1.04–1.11), but inversely associated with HNC (HR = 0.98 per 1 kg/m^2^ higher BMI, 95% CI 0.96–0.99). WHR was positively associated with the risk of both HNC (HR = 1.02 per 0.01 higher WHR, 95% CI 1.01–1.03) and OAC (HR = 1.06 per 0.01 higher WHR, 95% CI 1.04–1.08).

#### WHR as a mediator between UPF consumption and HNC risk

Only a small part of the positive association between UPF consumption (in %g/d) and HNC risk was mediated via WHR (5%, 95% CI 3–10%, *p* < 0.001; TNIE HR = 1.01 per 10% g/d higher UPF intake, 95% CI 1.01–1.01) (Table 2). Most of the association was not explained by WHR (PNDE HR = 1.22 per 10% g/d higher UPF intake, 95% CI 1.11–1.32). Furthermore, there was some evidence of an interaction between UPF consumption and WHR (*p*-value for interaction = 0.03).

#### WHR as a mediator between UPF consumption and OAC risk

The TE of UPF consumption (in %g/d) on OAC risk was decomposed into a PNDE of 1.17 (95% CI 0.99–1.33) and a TNIE via WHR of 1.03 (95% CI 1.02–1.03) (Table 2). Hence, the proportion mediated by WHR was 15% (95% CI 8–72%, *p* = 0.03) in the association with HNC.

#### BMI as a mediator between UPF consumption and OAC risk

Most of the association between UPF consumption (in %g/d) and higher OAC risk was not mediated via BMI (PNDE HR = 1.18 per 10% g/d higher UPF intake, 95% CI 1.00–1.34) (Table 2). BMI mediated 13% (95% CI 6–53%, *p* = 0.04) of the association (TNIE HR = 1.02 per 10% g/d higher UPF intake, 95% CI 1.01–1.03).

### Sensitivity analyses for the associations between ultra-processed food consumption and upper-aerodigestive tract cancers

Further adjusting for total water intake (including water in foods) or total energy intake produced similar results to those obtained in the main analyses (Supplementary Tables 7 and 8). Excluding participants censored in the first two years of follow-up (*N* = 442,536, since 7575 were excluded) did not substantially affect our results either (Supplementary Table 9). Likewise, the complete case analysis results (*N* = 419,590, of which 851 had HNC and 191 had OAC) and the results obtained using multiple imputation were similar to those obtained in our main analysis (Supplementary Tables 10 and 11).

Repeating the analyses using either the absolute intake of UPFs in grams per day (g/d) or the relative intake of UPFs in kilocalories per day (%kcal/d) as the exposure produced comparable results to those obtained in the main analysis (Supplementary Table 12 and Supplementary Fig. 9). Nevertheless, using the absolute intake of UPFs in kilocalories per day (kcal/d) as the exposure produced slightly different results to those in the main analysis, namely because UPF consumption was no longer associated with HNC risk (HR = 1.01 per 100 kcal/d higher UPF intake, 95% CI 0.99–1.03) (Supplementary Table 12 and Supplementary Fig. 9).

Lastly, we found a positive association between UPF consumption (%g/d) and accidental deaths (HR = 1.12 per 10% g/d higher UPF intake, 95% CI 1.02–1.23) in the negative control outcome analysis, after accounting for all the covariates included in the upper-aerodigestive tract cancer models (Supplementary Table 13). This association also withstood additional adjustments for BMI and type 2 diabetes mellitus (i.e. factors that may influence recovery after an accident) (results not shown).

## Discussion

In this large prospective cohort, UPF consumption (in %g/d) was associated with an increased risk of HNC and OAC. We did not find evidence of heterogeneity between HNC subtype association estimates. Furthermore, the positive association between UPF intake and HNC may be stronger in males than in females. Our negative control analysis suggests that at least part of the observed associations between the consumption of UPFs and the risk of upper-aerodigestive tract cancers is likely due to the influence of residual confounding. However, this does not necessarily mean that the associations are entirely non-causal; only that any causal estimate is likely smaller than we observed. In our mediation analysis, adiposity (i.e. BMI and WHR) only mediated a small proportion of the positive associations between UPF consumption and HNC and OAC.

Apart from the study conducted by Kliemann et al. [9] (which motivated our research), only one other study has investigated the associations between UPF consumption and HNC and OAC risk. Chang et al. [8] did not find an association between UPF consumption and the risk of HNC and OAC in the UK Biobank, in contrast to the findings in EPIC. A possible explanation for the null results in the UK Biobank could be limited power (197,426 UK Biobank participants of which 342 and 186, respectively, developed HNC and OAC over a median follow-up of 10 years, versus 450,111 EPIC participants of which 910 and 250, respectively, developed HNC and OAC over a median follow-up of 15 years). Residual confounding and the fact that the FFQs used were not designed to capture the extent and purpose of food processing may also partly explain the inconsistencies between studies.

Our mediation results are in line with existing findings. First, UPFs have been associated with excess weight (i.e. obesity and BMI) and central adiposity (i.e. WHR and waist circumference) in several observational studies [10–13]. UPFs are highly palatable energy-dense foods with low nutritional quality. They are convenient, cheap, and often sold in large portions [4, 54]. This, in addition to their reduced satiety potential [55], favours the consumption of large portions and an excessive amount of calories. Some studies even suggest that the consumption of UPFs may disrupt the gut microbiota, induce inflammation, and cause endocrine changes that disturb energy balance and increase the risk of obesity [11, 56, 57]. Second, multiple studies suggest that excess weight and abdominal obesity are positively associated with OAC risk[15–20, 58], and that central adiposity may be a risk factor for HNC [21, 22]. Hence, it is plausible that BMI and WHR mediate the association between UPF consumption and OAC risk, and that WHR mediates the association between UPF consumption and HNC risk. Notwithstanding, our findings indicate that the mediated effects via BMI and WHR are small and that other mechanisms are likely involved.

A review of prospective cohort studies suggested that diet quality did not play an important role in the positive associations between UPF consumption, obesity and obesity-related outcomes (e.g. cancer), since the adjustment for several dietary factors did not substantially attenuate the associations [59]. The authors argue that ultra-processing itself may be associated with disease risk, independent of nutritional quality. This has profound implications for the food industry, as it could mean that UPF reformulation would not be sufficient to tackle the risks associated with UPF consumption. We acknowledge that this may just be another case of ‘highly consequential but misleading findings’ [60], where the observed associations are not causal but rather an artefact of residual confounding [61, 62] (the association of UPF consumption with accidental death in EPIC provides some support for this interpretation). However, if these associations truly reflect causality, the presence of carcinogenic compounds in UPFs, such as neo-formed contaminants produced during heat treatment, contaminants transferred from packaging materials and additives used to preserve and improve the organoleptic properties of food [5] may partly explain the relation between UPF intake and upper-aerodigestive tract cancer risk.

In sensitivity analyses, we did not find an association between a 100 kcal/d higher UPF consumption and HNC risk. This could be because the consumption of artificially sweetened UPFs (which may contain potentially carcinogenic compounds like aspartame and 4-methylimidazole [63]) is likely disregarded or underestimated when using kcal/d as a measure of UPF intake. We acknowledge that this is an arguable hypothesis since Chazelas et al. [64] did not find an association between artificially sweetened drinks and cancer in the NutriNet-Santé cohort. Nevertheless, a study in the same cohort found a positive association between higher artificial sweetener intake from all food sources and cancer risk, suggesting other artificially sweetened UPFs (e.g. yoghurts, breakfast cereals, gelatine desserts) may play a role in cancer incidence [65].

Some of the strengths of this study include the large sample size of the EPIC cohort and its long follow-up time. The prospective nature of EPIC and the availability of measured rather than self-reported BMI and WHR were also advantages. Moreover, EPIC’s multi-centre design increases the diversity of our study sample. Another advantage is that cancer cases were detected through registries (which provide detailed information on cancer subtypes) and active follow-up methods, both of which are unlikely to be affected by measurement error. Finally, the use of several measures of UPF intake (%g/d, g/d, %kcal/d and kcal/d) as the exposure makes our results more comparable to previous studies investigating the associations between the consumption of UPFs and cancer [5–9].

We acknowledge that this study has several limitations. For instance, in our mediation analysis we assumed that the associations between UPF consumption and upper-aerodigestive tract cancers were not influenced by residual confounding or measurement error. These are strong assumptions since residual confounding due to unmeasured (e.g. human papilloma-virus infection) or imprecisely measured (e.g. smoking and alcohol intake) confounders inevitably biased our estimates to some extent (since estimates changed when the models were adjusted for potential confounders for which some values were missing or likely measured with error). Indeed, the fact that our negative control outcome analysis suggested that UPF consumption may be associated with accidental deaths points to the possibility of residual confounding.

For our mediation analysis, we used data on BMI and WHR collected at baseline. Admittedly, a limitation with this approach is that the exposure data were not gathered prior to the mediator data, so we cannot be certain that the exposure temporally precedes the mediator. Follow-up data on measured BMI and WHR were only available for 5% and 27% of the participants who answered the lifestyle follow-up questionnaire (*N* = 349,283), respectively. Unfortunately, cancer cases among participants with complete follow-up data were insufficient for us to conduct any sensitivity analyses using follow-up data.

An additional limitation is that we assumed that BMI and WHR mediated the association between UPF consumption and OAC risk through separate pathways. Since BMI and WHR are correlated, it would be incorrect to assume that the proportion mediated via adiposity equals the sum of the proportions mediated through both BMI and WHR. Therefore, in this association, the proportion mediated via adiposity is likely less than 28% (13% via BMI plus 15% via WHR).

Another issue is that the FFQs used at baseline were not designed to distinguish between NOVA groups [66], potentially leading to random misclassification bias and the weakening of our association estimates. Nonetheless, Huybrechts et al. [31] found positive correlations between UPF consumption and food processing biomarkers (i.e. plasma elaidic acid, an unsaturated trans-fatty acid, and urinary 4-methylsyringol sulphate) in EPIC, suggesting that UPFs were likely correctly identified in the dataset. Also, the dietary data used in our analyses were collected only once, at baseline in the 1990s, when the availability and consumption of UPFs was relatively lower than today. Hence, this study relies on the somewhat unrealistic assumption that UPF intake was rather low and did not increase over time [67]. When Kliemann et al. [9] explored the association between the consumption of UPFs and cancer in EPIC under a hypothetical “upper bound scenario” (where foods were classified into NOVA groups based on their highest degree of processing possible), results were similar to those obtained under the more conservative “middle bound scenario” used in our study (where foods were classified into NOVA groups based on their most likely degree of processing at the time of dietary data collection). Although the use of a hypothetical “upper bound scenario” may account for some of the changes in dietary intake during follow-up, regression dilution bias was still an issue Kliemann et al. [9] could not account for in their analyses. Consequently, dietary questionnaire-related biases may have led to the underestimation of the association between UPF consumption and cancer in the EPIC cohort.

In conclusion, we reaffirmed that UPF intake is associated with an increased risk of HNC and OAC in the EPIC study. Since BMI and WHR explain little of the associations between UPF consumption and upper-aerodigestive tract cancers, further research is required to investigate other mechanisms that may be at play (if there is indeed any causal effect of UPF consumption on these cancers). Our results are likely influenced by residual confounding, as indicated by the negative control analysis. Therefore, our findings should be regarded with caution until they are replicated in other settings (i.e. in populations with different underlying confounding structures) or triangulated with evidence obtained using other methodological approaches.

## Supplementary Material

Supplementary Material

## Figures and Tables

**Table 1:** Baseline characteristics of study participants by sex-specific quartiles of relative ultra-processed food consumption (%g/d)

	Q1 (M: ≤ 8.95%g/d; F: ≤ 6.94%g/d)	Q2 (M: 8.96–13.16%g/d; F: 6.95–11.30%g/d)	Q3 (M: 13.17–18.62%g/d; F: 11.31–17.54%g/d)	Q4 (M: 18.63–85.16%g/d; F: 17.55–90.53%g/d)	Overall
Participants, *n*	112,529	112,528	112,527	112,527	450,111
Age at recruitment (years), mean ± SD	52.9 ± 7.74	52.5 ± 8.87	51.1 ± 10.2	48.1 ± 11.1	51.1 ± 9.75
Females, *n* (%)	79,672 (70.8)	79,672 (70.8)	79,671 (70.8)	79,671 (70.8)	318,686 (70.8)
Country, *n* (%)					
France	38,677 (34.4)	19,085 (17.0)	7592 (6.7)	2049 (1.8)	67,403 (15.0)
Italy	17,500 (15.6)	13,098 (11.6)	8935 (7.9)	5012 (4.5)	44,545 (9.9)
Spain	23,830 (21.2)	8159 (7.3)	4975 (4.4)	3025 (2.7)	39,989 (8.9)
United Kingdom	2987 (2.7)	13,407 (11.9)	26,794 (23.8)	32,228 (28.6)	75,416 (16.8)
The Netherlands	3217 (2.9)	10,035 (8.9)	13,335 (11.9)	9951 (8.8)	36,538 (8.1)
Germany	6324 (5.6)	11,200 (10.0)	14,136 (12.6)	16,897 (15.0)	48,557 (10.8)
Sweden	8854 (7.9)	16,577 (14.7)	14,299 (12.7)	8944 (7.9)	48,674 (10.8)
Denmark	10,934 (9.7)	19,621 (17.4)	14,384 (12.8)	10,075 (9.0)	55,014 (12.2)
Norway	206 (0.2)	1346 (1.2)	8077 (7.2)	24,346 (21.6)	33,975 (7.5)
Height^a^ (cm), mean ± SD	164 ± 8.65	166 ± 8.99	167 ± 8.83	168 ± 8.57	166 ± 8.86
Measured BMI^b^ (kg/m^2^), mean ± SD	26.2 ± 4.25	25.5 ± 4.10	25.4 ± 4.12	25.5 ± 4.41	25.6 ± 4.22
Missing, *n* (%)	27,851 (24.8)	15,490 (13.8)	14,348 (12.8)	26,873 (23.9)	84,562 (18.8)
Measured WHR^c^, mean ± SD	0.86 ± 0.1	0.84 ± 0.1	0.83 ± 0.1	0.84 ± 0.1	0.84 ± 0.1
Missing, *n* (%)	31,561 (28.0)	24,491 (21.8)	23,256 (20.7)	33,945 (30.2)	113,253 (25.2)
Education level, *n* (%)					
None	8622 (7.7)	3120 (2.8)	2190 (1.9)	1619 (1.4)	15,551 (3.5)
Primary school completed	30,898 (27.5)	28,771 (25.6)	26,533 (23.6)	24,862 (22.1)	111,064 (24.7)
Technical/Professional school	14,072 (12.5)	24,757 (22.0)	30,300 (26.9)	34,653 (30.8)	103,782 (23.1)
Secondary school	28,716 (25.5)	23,685 (21.0)	20,675 (18.4)	20,834 (18.5)	93,910 (20.9)
Higher education	27,810 (24.7)	28,964 (25.7)	27,535 (24.5)	24,622 (21.9)	108,931 (24.2)
Missing	2411 (2.1)	3231 (2.9)	5294 (4.7)	5937 (5.3)	16,873 (3.7)
Physical activity level, *n* (%)					
Inactive	27,550 (24.5)	21,938 (19.5)	20,355 (18.1)	18,189 (16.2)	88,032 (19.6)
Moderately inactive	39,820 (35.4)	38,982 (34.6)	36,912 (32.8)	34,227 (30.4)	149,941 (33.3)
Moderately active	27,477 (24.4)	28,369 (25.2)	29,751 (26.4)	34,602 (30.8)	120,199 (26.7)
Active	17,185 (15.3)	21,700 (19.3)	22,470 (20.0)	21,760 (19.3)	83,115 (18.5)
Missing	497 (0.4)	1539 (1.4)	3039 (2.7)	3749 (3.3)	8824 (2.0)
Smoking status, *n* (%)					
Never	57,091 (50.7)	54,677 (48.6)	53,788 (47.8)	53,738 (47.8)	219,294 (48.7)
Former	28,412 (25.2)	31,268 (27.8)	32,246 (28.7)	30,754 (27.3)	122,680 (27.3)
Current	24,549 (21.8)	24,830 (22.1)	24,684 (21.9)	25,651 (22.8)	99,714 (22.2)
Missing	2477 (2.2)	1753 (1.6)	1809 (1.6)	2384 (2.1)	8423 (1.9)
Alcohol intake (g/d), mean ± SD	15.3 ± 21.1	13.0 ± 17.1	10.7 ± 14.4	8.00 ± 12.3	11.7 ± 16.8
Energy intake (kcal/d), mean ± SD	2100 ± 622	2110 ± 598	2140±607	2200±666	2140±625
Carbohydrate intake (g/d), mean ± SD	237 ± 75.7	245 ± 72.3	257 ± 75.6	278 ± 91.6	254 ± 80.7
Fat intake (g/d), mean ± SD	79.4 ± 28.2	81.4 ± 28.6	82.7 ± 29.8	82.6 ± 31.7	81.5 ± 29.6
Fibre intake (g/d), mean ± SD	24.3 ± 8.74	23.3 ± 8.10	23.8 ± 8.54	23.9 ± 9.04	23.8 ± 8.62
Sodium intake (mg/d), mean ± SD	2500 ± 968	2650 ± 1110	2630 ± 1190	2650±1290	2610 ± 1150
Relative intake of ultra-processed foods (%g/d), mean ± SD	4.95 ± 1.90	9.65 ± 1.53	14.6 ± 1.85	25.7 ± 7.78	13.7 ± 8.76
Absolute intake of ultra-processed foods (g/d), mean ± SD	141 ± 80.1	273±104	393 ± 138	648 ± 321	364 ± 264
Relative intake of processed foods (%g/d), mean ± SD	16.9 ± 12.0	14.2 ± 10.0	12.5 ± 8.85	10.7 ± 7.43	13.6 ± 10.0
Absolute intake of processed foods (g/d), mean ± SD	445 ± 367	385 ± 313	328±270	271 ± 237	357 ± 308
Relative intake of unprocessed/minimally processed foods (%g/d), mean ± SD	76.6 ± 12.7	74.9 ± 10.7	71.9 ± 9.50	62.7 ± 10.3	71.5 ± 12.1
Absolute intake of unprocessed/minimally processed foods (g/d), mean ± SD	2190±985	2130 ± 818	1940±699	1590 ± 653	1960 ± 833

Baseline characteristics stratified by sex-specific quartiles of relative intake of ultra-processed foods (%g/d). Abbreviations: BMI, Body mass index; WHR, Waist-to-hip ratio; Q1, quartile 1; Q2, quartile 2; Q3, quartile 3; Q4, quartile 4; M, Males; F, Females; SD, standard deviation

a When measured/self-reported height values were not available, missing values were imputed with mean centre-, age- and sex-specific height values

b According to the World Health Organization [53], a healthy adult BMI lies between 18.5 and 24.9 kg/m^2^

c According to the World Health Organization [53], a healthy WHR corresponds to < 0.9 for males and < 0.85 for females

**Table 2:** Mediation analysis for the associations between ultra-processed food consumption (in %g/d) and head and neck cancer and oesophageal adenocarcinoma, where the potential mediators (i.e. waist-to-hip ratio and body mass index) were measured at baseline

Association	Mediator	Effect	Estimate	95% CI	*P*-value	*P*-value for interaction
Ultra-processed food consumption and head and neck cancer risk	Waist-to-hip ratio	TE, HR	1.23	1.12–1.33	< 0.001	0.03
		PNDE, HR	1.22	1.11–1.32	< 0.001	
		TNIE, HR	1.01	1.01–1.01	< 0.001	
		PM, %	5.32	2.73–10.27	< 0.001	
Ultra-processed food consumption and oesophageal adenocarcinoma risk	Waist-to-hip ratio	TE, HR	1.20	1.02–1.36	0.032	0.38
		PNDE, HR	1.17	0.99–1.33	0.06	
		TNIE, HR	1.03	1.02–1.03	< 0.001	
		PM, %	14.85	7.57–72.27	0.03	
	Body mass index	TE, HR	1.21	1.02–1.37	0.038	0.28
		PNDE, HR	1.18	1.00–1.34	0.06	
		TNIE, HR	1.02	1.01–1.03	< 0.001	
		PM, %	13.38	5.69–52.86	0.04	

Point estimates were obtained by direct counterfactual imputation estimation and confidence intervals were obtained using 1000 bootstrap repetitions. Results were scaled to reflect a 10% g/d higher consumption of ultra-processed foods. Mediation models were adjusted for age at recruitment in 1-year categories, sex, sub-centre, education, physical activity, height, smoking status and alcohol intake. The total effect corresponds to the multiplication of the pure natural direct effect and the total natural indirect effect. Waist-to-hip ratio models included 336,858 participants, of which 828 and 195 had head and neck cancer and oesophageal adenocarcinoma, respectively. Body mass index models included 365,549 participants, of which 212 had oesophageal adenocarcinoma. *HR* hazard ratio; *CI* confidence interval; *TE* total effect in presence of the mediator; *PNDE* pure natural direct effect; *TNIE* total natural indirect effect; *PM* proportion mediated

**Table 3:** STROBE statement—checklist of items that should be included in reports of observational studies

	Item no	Recommendation	Page no	Relevant text from manuscript
Title and abstract	1	(*a*) Indicate the study’s design with a commonly used term in the title or the abstract	3	“European Prospective Investigation into Cancer and Nutrition (EPIC) cohort”
		(*b*) Provide in the abstract an informative and balanced summary of what was done and what was found	3	“We used Cox regressions to investigate the associations…”
*Introduction*				
Background/rationale	2	Explain the scientific background and rationale for the investigation being reported	4	“In recent years, there has been growing interest … this has not been investigated using mediation analysis”
Objectives	3	State specific objectives, including any prespecified hypotheses	4–5	“The aim of this study was…”
*Methods*				
Study design	4	Present key elements of study design early in the paper	6	“The EPIC cohort” subsection
Setting	5	Describe the setting, locations, and relevant dates, including periods of recruitment, exposure, follow-up, and data collection	6	“The EPIC cohort” and “Study sample” subsections
Participants	6	(*a*) *Cohort study*—Give the eligibility criteria, and the sources and methods of selection of participants. Describe methods of follow-up *Case–control study*—Give the eligibility criteria, and the sources and methods of case ascertainment and control selection. Give the rationale for the choice of cases and controls *Cross-sectional study*—Give the eligibility criteria, and the sources and methods of selection of participants	6–7	“Ascertainment of cancer cases” and “Study sample” subsections
		(*b*) *Cohort study*—For matched studies, give matching criteria and number of exposed and unexposed *Case–control study*—For matched studies, give matching criteria and the number of controls per case	NA	NA
Variables	7	Clearly define all outcomes, exposures, predictors, potential confounders, and effect modifiers. Give diagnostic criteria, if applicable	6–8	“Dietary data and food processing variables”, “Ascertainment of cancer cases”, “Covariates” and “Potential mediators” subsections
Data sources/measurement	8*	For each variable of interest, give sources of data and details of methods of assessment (measurement). Describe comparability of assessment methods if there is more than one group	6–8	“Dietary data and food processing variables”, “Ascertainment of cancer cases”, “Covariates” and “Potential mediators” subsections
Bias	9	Describe any efforts to address potential sources of bias	9	“Model 1 was stratified by age at recruitment in 1-year categories, sex, and sub-centre. Model 2 was additionally adjusted for education, physical activity, height, and smoking status. Model 3 was additionally adjusted for alcohol intake in g/d to reflect the association between the consumption of foods by their degree of processing and cancer, regardless of alcohol intake”
Study size	10	Explain how the study size was arrived at	6	“Study sample” subsection
Quantitative variables	11	Explain how quantitative variables were handled in the analyses. If applicable, describe which groupings were chosen and why	8–12	“Covariates”, “Potential mediators” and “Statistical analysis” subsections
Statistical methods	12	(*a*) Describe all statistical methods, including those used to control for confounding	8–12	“Statistical analysis” subsection
		(*b*) Describe any methods used to examine subgroups and interactions	9	“… we stratified Model 3 (for every exposure-outcome combination)”
		(*c*) Explain how missing data were addressed	9	“We used single-value imputation to deal with missing data among covariates used to control for potential confounding…”
		(*d*) *Cohort study*—If applicable, explain how loss to follow-up was addressed *Case–control study*—If applicable, explain how matching of cases and controls was addressed *Cross-sectional study*—If applicable, describe analytical methods taking account of sampling strategy	9	“…time of exit was defined as age at first cancer diagnosis (excluding non-melanoma skin cancer) or age at last follow-up (i.e. death, emigration, loss to follow-up or end of follow-up [i.e. between June 2008 and December 2013, depending on the centre]), whichever came first”
		(*e*) Describe any sensitivity analyses	11	“Sensitivity analyses” within the “Statistical analysis” subsection
*Results*				
Participants	13*	(a) Report numbers of individuals at each stage of study—e.g. numbers potentially eligible, examined for eligibility, confirmed eligible, included in the study, completing follow-up, and analysed	6	“Study sample” subsection in “Methods” section
		(b) Give reasons for non-participation at each stage	NA	NA
		(c) Consider use of a flow diagram	Supplement	Supplementary Fig. 1. Flowchart of European Prospective Investigation into Cancer and Nutrition (EPIC) participants included in the study
Descriptive data	14*	(a) Give characteristics of study participants (e.g. demographic, clinical, social) and information on exposures and potential confounders	13–14	“Descriptive characteristics” subsection
		(b) Indicate number of participants with missing data for each variable of interest	13–14	“Descriptive characteristics” subsection
		(c) *Cohort study*—Summarise follow-up time (e.g. average and total amount)	14	“During a mean follow-up of 14.13±3.98 years (6,358,569 person-years, median follow-up = 14.95 years; range 1 day to 22.79 years)”
Outcome data	15*	*Cohort study*—Report numbers of outcome events or summary measures over time *Case–control study*—Report numbers in each exposure category, or summary measures of exposure *Cross-sectional study*—Report numbers of outcome events or summary measures	14	“During a mean follow-up of 14.13±3.98 years (6.358.569 person-years. median follow-up = 14.95 years; range 1 day to 22.79 years)”
Main results	16	(*a*) Give unadjusted estimates and, if applicable, confounder-adjusted estimates and their precision (e.g. 95% confidence interval). Make clear which confounders were adjusted for and why they were included	14–18	“Associations between the consumption of ultra-processed foods and upper-aerodigestive tract cancers” and “Mediating role of adiposity in the associations between ultra-processed food consumption and upper-aerodigestive tract cancers” subsections
		(*b*) Report category boundaries when continuous variables were categorised	9	“Furthermore, we stratified Model 3 (for every exposure-outcome combination) by alcohol intake (as defined by Wozniak et al.[45], i.e. no/light alcohol intake [0.1–6 g/d (men); 0.1–3 g/d (women)], moderate alcohol intake [6.1–24 g/d (men); 3.1–24 g/d (women)], heavy alcohol intake [>24 g/d])”
		(*c*) If relevant, consider translating estimates of relative risk into absolute risk for a meaningful time period	NA	NA
Other analyses	17	Report other analyses done—e.g. analyses of subgroups and interactions, and sensitivity analyses	14–18	“Associations between the consumption of ultra-processed foods and upper-aerodigestive tract cancers”, “Mediating role of adiposity in the associations between ultra-processed food consumption and upper-aerodigestive tract cancers” and “Sensitivity analyses for the associations between ultra-processed food consumption and upper-aerodigestive tract cancers” subsections
*Discussion*				
Key results	18	Summarise key results with reference to study objectives	19	“In this large prospective cohort, UPF consumption (in %g/d) was associated with…”
Limitations	19	Discuss limitations of the study, taking into account sources of potential bias or imprecision. Discuss both direction and magnitude of any potential bias	20–21	“We acknowledge that this study has several limitations… dietary questionnaire-related biases may have led to the underestimation of the association between UPF consumption and cancer in the EPIC cohort.”
Interpretation	20	Give a cautious overall interpretation of results considering objectives, limitations, multiplicity of analyses, results from similar studies, and other relevant evidence	19–21	“Discussion” section
Generalisability	21	Discuss the generalisability (external validity) of the study results	19–21	“Discussion” section
*Other information*				
Funding	22	Give the source of funding and the role of the funders for the present study and, if applicable, for the original study on which the present article is based	23	“Funding” subsection in the “Statements & Declarations” section

Note: An Explanation and Elaboration article discusses each checklist item and gives methodological background and published examples of transparent reporting. The STROBE checklist is best used in conjunction with this article (freely available on the Web sites of PLoS Medicine at http://www.plosmedicine.org/, Annals of Internal Medicine at http://www.annals.org/, and Epidemiology at http://www.epidem.com/). Information on the STROBE Initiative is available at www.strobe-statement.org

* Give information separately for cases and controls in case–control studies and, if applicable, for exposed and unexposed groups in cohort and cross-sectional studies

## Data Availability

The data described in the manuscript will be made available upon request pending application and approval. The analytic code will be made publicly and freely available without restriction at https://github.com/fernandam93/UPF_adiposity_UADT.

## Abbreviations

BMI: Body mass index
CI: Confidence interval
EPIC: European Prospective Investigation into Cancer and Nutrition
FFQ: Food frequency questionnaire
HNC: Head and neck cancer
HR: Hazard ratio
IARC: International Agency for Research on Cancer
INHANCE: International Head and Neck Cancer Epidemiology
MICE: Multivariate imputation by chained equations
OAC: Oesophageal adenocarcinoma
PM: Proportion mediated
PNDE: Pure natural direct effect
SD: Standard deviation
TE: Total effect
TNIE: Total natural indirect effect
UPF: Ultra-processed food
WHR: Waist-to-hip ratio
