# Supplementary material for: Ultra-processed foods, adiposity and risk of head and neck cancer and oesophageal adenocarcinoma in the European Prospective Investigation into Cancer and Nutrition study: a mediation analysis
Source: Eur J Nutr. Author manuscript; Available in PMC 2024 Mar 4. (PMC10899298; doi:10.1007/s00394-023-03270-1)
Supplement: Supplementary Material [file EMS192895-supplement-Supplementary_Material.pdf]

Supplementary Material

Ultra-processed foods, adiposity and risk of head and neck cancer and oesophageal adenocarcinoma in the European Prospective Investigation into Cancer and Nutrition study: a mediation analysis

Fernanda Morales-Berstein[1,2], Carine Biessy[3], Vivian Viallon[3], Ana Goncalves-Soares[1,2], Corinne Casagrande[3], Bertrand Hémon[3], Nathalie Kliemann[3,4], Manon Cairat[3,5], Jessica Blanco Lopez[3], Aline Al Nahas[3], Kiara Chang[6], Eszter Vamos[6], Fernanda Rauber[7,8], Renata Bertazzi Levy[7,8], Diana Barbosa Cunha[9], Paula Jakszyn[10-12], Pietro Ferrari[3], Paolo Vineis[13,14], Giovanna Masala[15], Alberto Catalano[16], Emily Sonestedt[17], Yan Borné[17], Verena Katzke[18], Rashmita Bajracharya[18], Claudia Agnoli[19], Marcela Guevara[20-22], Alicia Heath[23], Loredana Radoï[5], Francesca Mancini[5], Elisabete Weiderpass[24], José María Huerta[25,21], María-José Sánchez[26-28,21], Anne Tjønneland[29,30], Cecilie Kyrø[29], Matthias B Schulze[31,32], Guri Skeie[33], Marko Lukic[33], Tonje Braaten[33], Marc Gunter[23,3], Christopher Millett[6,34], Antonio Agudo[10,11], Paul Brennan[35], M Carolina Borges[1,2], Rebecca C Richmond[1,2], Tom G Richardson[1,2], George Davey Smith[1,2], Caroline L Relton[1,2], Inge Huybrechts[3], on behalf of the EPIC Network

[1]MRC Integrative Epidemiology Unit, University of Bristol, Bristol, United Kingdom.
[2]Population Health Sciences, Bristol Medical School, University of Bristol, Bristol, United Kingdom.
[3]Nutrition and Metabolism Branch, International Agency for Research on Cancer/World Health Organization, Lyon, France.
[4]Cancer Research Center of Santa Catarina, CEPON, Florianópolis, Brazil.
[5]Paris-Saclay University, UVSQ, Inserm "Exposome, heredity, cancer and health" team, CESP U1018, Gustave Roussy, Villejuif, France.
[6]Public Health Policy Evaluation Unit, School of Public Health, Imperial College London, London, United Kingdom.
[7]Preventive Medicine Department of the Medical School, University of São Paulo, São Paulo, Brazil.
[8]Center for Epidemiological Research in Nutrition and Health, University of São Paulo, São Paulo, Brazil.
[9]Hésio Cordeiro Institute of Social Medicine, Department of Epidemiology, Rio de Janeiro State University, Rio de Janeiro, RJ, Brazil.
[10]Unit of Nutrition and Cancer, Catalan Institute of Oncology-ICO, L'Hospitalet de Llobregat, Spain.
[11]Nutrition and Cancer Group; Epidemiology, Public Health, Cancer Prevention and Palliative Care Program; Bellvitge Biomedical Research Institute - IDIBELL, L'Hospitalet de Llobregat, Spain.
[12]Blanquerna Faculty of Health Sciences, Ramon Llull University, Barcelona, Spain
[13]MRC Centre for Environment and Health, School of Public Health, Imperial College London, Norfolk Place, W2 1PG London, UK.
[14]Italian Institute of Technology, Genova, Italy.
[15]Institute for Cancer Research, Prevention and Clinical Network (ISPRO), Florence Italy.
[16]Centre for Biostatistics, Epidemiology, and Public Health, Department of Clinical and Biological Sciences, University of Turin, 10043 Orbassano (TO), Italy.

[17]*Nutrition Epidemiology, Department of Clinical Sciences Malmö, Faculty of Medicine, Lund University, Lund, Sweden.*
[18]*Department of Cancer Epidemiology, German Cancer Research Center (DKFZ), Heidelberg, Germany.*
[19]*Epidemiology and Prevention Unit, Fondazione IRCCS Istituto Nazionale dei Tumori, Milan, Italy.*
[20]*Instituto de Salud Pública y Laboral de Navarra, 31003 Pamplona, Spain.*
[21]*Centro de Investigación Biomédica en Red de Epidemiología y Salud Pública (CIBERESP), 28029 Madrid, Spain.*
[22]*Navarra Institute for Health Research (IdiSNA), 31008 Pamplona, Spain.*
[23]*Department of Epidemiology and Biostatistics, School of Public Health, Imperial College London, London, United Kingdom.*
[24]*International Agency for Research on Cancer, World Health Organization, Lyon, France.*
[25]*Department of Epidemiology, Murcia Regional Health Council-IMIB, Murcia, Spain.*
[26]*Escuela Andaluza de Salud Pública (EASP), 18011 Granada, Spain.*
[27]*Instituto de Investigación Biosanitaria ibs.GRANADA, 18012 Granada, Spain.*
[28]*Department of Preventive Medicine and Public Health, University of Granada, 18071 Granada, Spain.*
[29]*Danish Cancer Society Research Center, Diet, Cancer and Health, Strandboulevarden 49, DK-2100 Copenhagen, Denmark.*
[30]*Department of Public Health, University of Copenhagen, DK-2200 Copenhagen, Denmark.*
[31]*Department of Molecular Epidemiology, German Institute of Human Nutrition Potsdam-Rehbruecke, Nuthetal, Germany.*
[32]*Institute of Nutritional Science, University of Potsdam, Nuthetal, Germany.*
[33]*Department of Community Medicine, Faculty of Health Sciences, UiT The Arctic University of Norway, Tromsø, Norway.*
[34]*NOVA National School of Public Health, Public Health Research Centre, Comprehensive Health Research Center, CHRC, NOVA University Lisbon, Lisbon, Portugal.*
[35]*Genetic Epidemiology Group, International Agency for Research on Cancer, World Health Organization, Lyon, France.*

For correspondence: dy20206@bristol.ac.uk

Supplementary Figures

1. Flowchart of European Prospective Investigation into Cancer and Nutrition (EPIC) participants included in the study.
2. Histogram of the relative intake of ultra-processed foods in grams per day.
3. Log-log survival plot for ultra-processed food consumption and head and neck cancer risk.
4. Log-log survival plot for ultra-processed food consumption and oesophageal adenocarcinoma risk.
5. Schoenfeld residuals for the association between ultra-processed food consumption and head and neck cancer risk.
6. Schoenfeld residuals for the association between ultra-processed food consumption and oesophageal adenocarcinoma risk.
7. Correlation plot for ultra-processed food consumption models.
8. Non-linearity assessment for the relative intake of ultra-processed foods in grams per day and head and neck cancer risk.
9. Forest plots for associations between the intake of ultra-processed foods (in %g/d, g/d, %kcal/d and kcal/d) and the risk of head and neck cancer and oesophageal adenocarcinoma.

Supplementary Tables

1. Food categories contributing to each NOVA group.
2. Variance inflation factor (VIF) for the assessment of multicollinearity.
3. Associations between the relative intake of ultra-processed foods (in %g/d) and the risk of head and neck cancer and oesophageal adenocarcinoma.
4. Associations between the relative intake of ultra-processed foods (in %g/d) and the risk of head and neck cancer subtypes.
5. Stratified analyses for the associations between ultra-processed foods (in %g/d) and the risk of head and neck cancer and oesophageal adenocarcinoma.
6. Identification of possible mediators of the associations between ultra-processed food consumption (in %g/d) and the risk of head and neck and oesophageal adenocarcinoma.
7. Associations between the relative intake of ultra-processed foods (in %g/d) and the risk of head and neck cancer and oesophageal adenocarcinoma, accounting for total water intake (including water in foods).
8. Associations between relative intake of ultra-processed foods (in %g/d) and the risk of head and neck cancer and oesophageal adenocarcinoma, accounting for energy intake.
9. Associations between the relative intake of ultra-processed foods (in %g/d) and the risk of head and neck cancer and oesophageal adenocarcinoma, excluding participants censored in the first two years of follow-up.
10. Complete case analysis for the associations between the relative intake of ultra-processed foods (in %g/d) and the risk of head and neck cancer and oesophageal adenocarcinoma.
11. Multivariate imputation by chained equations analyses for the associations between the relative intake of ultra-processed foods (in %g/d) and the risk of head and neck cancer and oesophageal adenocarcinoma.

12. Associations between the absolute (in g/d and kcal/d) and relative intake (in %kcal/d) of ultra-processed foods and the risk of head and neck cancer and oesophageal adenocarcinoma.
13. Negative control outcome analysis for the association between the relative intake of ultra-processed foods (in %g/d) and the risk of accidental deaths.

Supplementary Figure 1. Flowchart of European Prospective Investigation into Cancer and Nutrition (EPIC) participants included in the study.

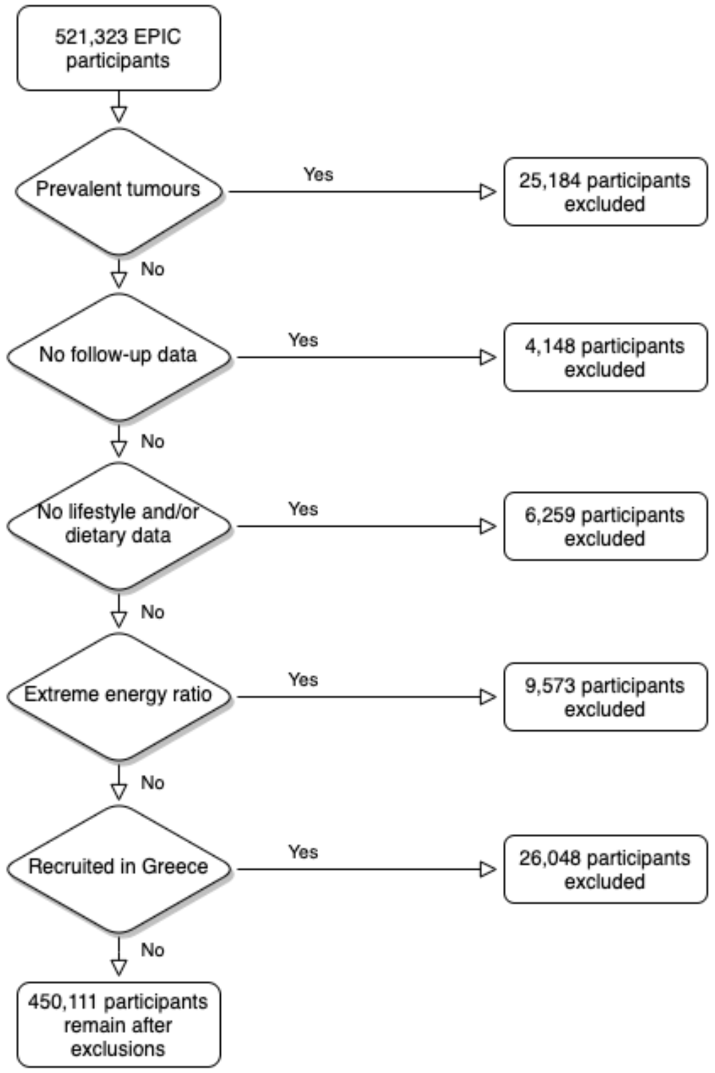

Supplementary Figure 2. Histogram of the relative intake of ultra-processed foods in grams per day.

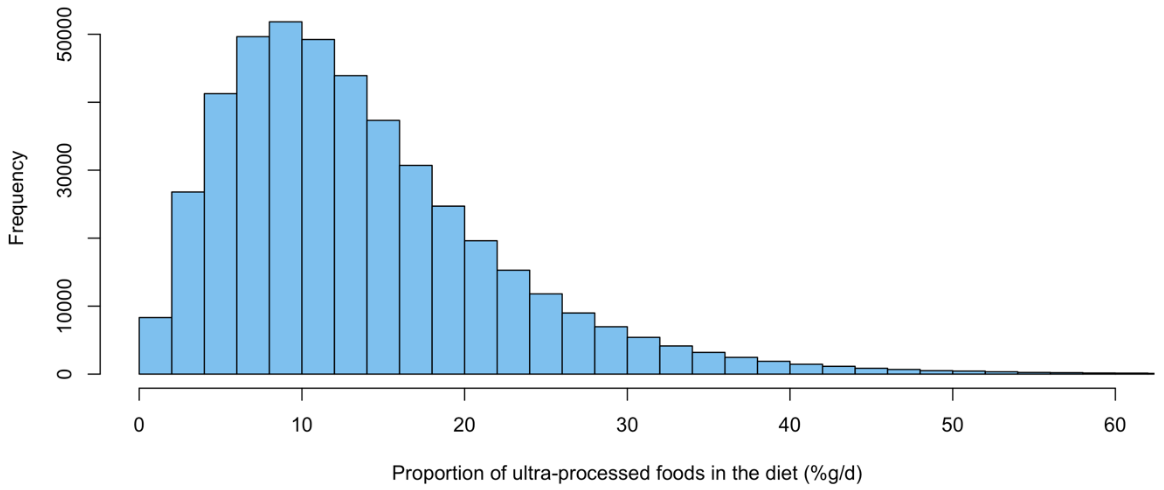

Min.	1st Qu.	Median	Mean	3rd Qu.	Max.
0.000	7.473	11.912	13.713	17.895	90.533

Supplementary Figure 3. Log-log survival plot for ultra-processed food consumption and head and neck cancer risk.

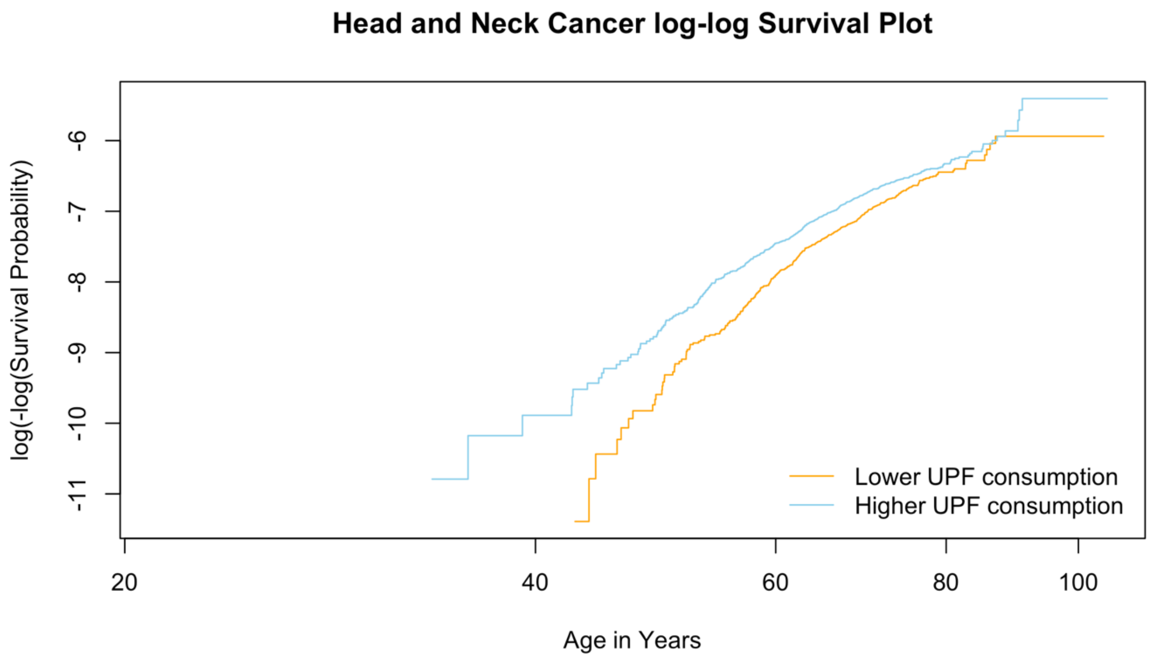

Supplementary Figure 4. Log-log survival plot for ultra-processed food consumption and oesophageal adenocarcinoma risk.

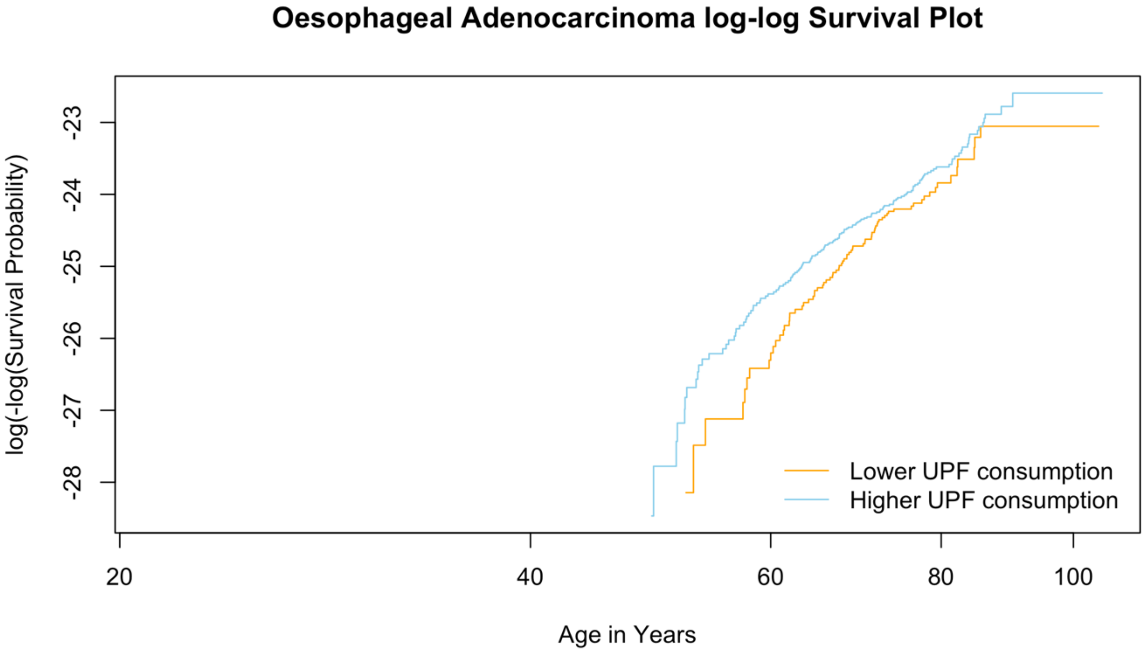

Supplementary Figure 5. Schoenfeld residuals for the association between ultra-processed food consumption and head and neck cancer risk.

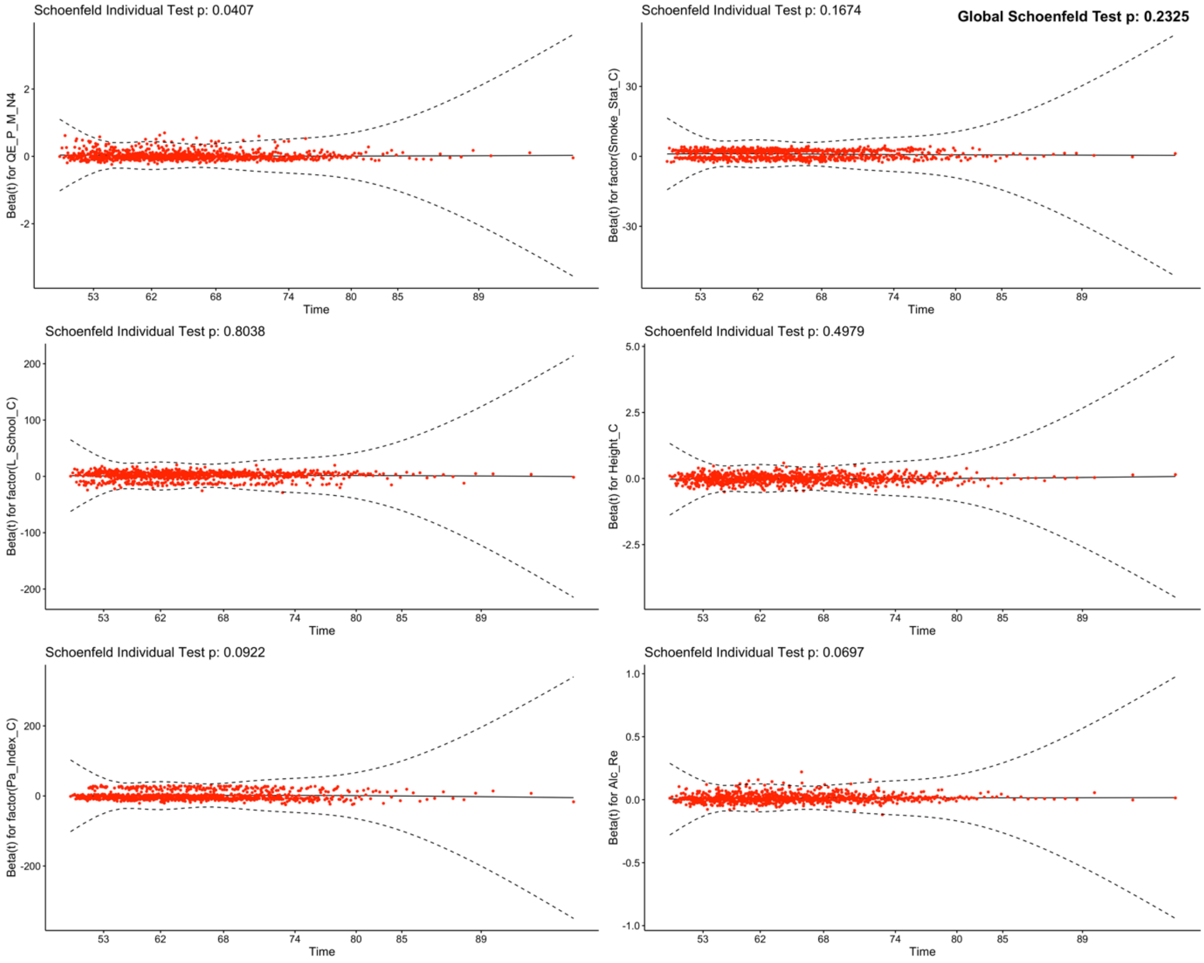

Supplementary Figure 6. Schoenfeld residuals for the association between ultra-processed food consumption and oesophageal adenocarcinoma risk.

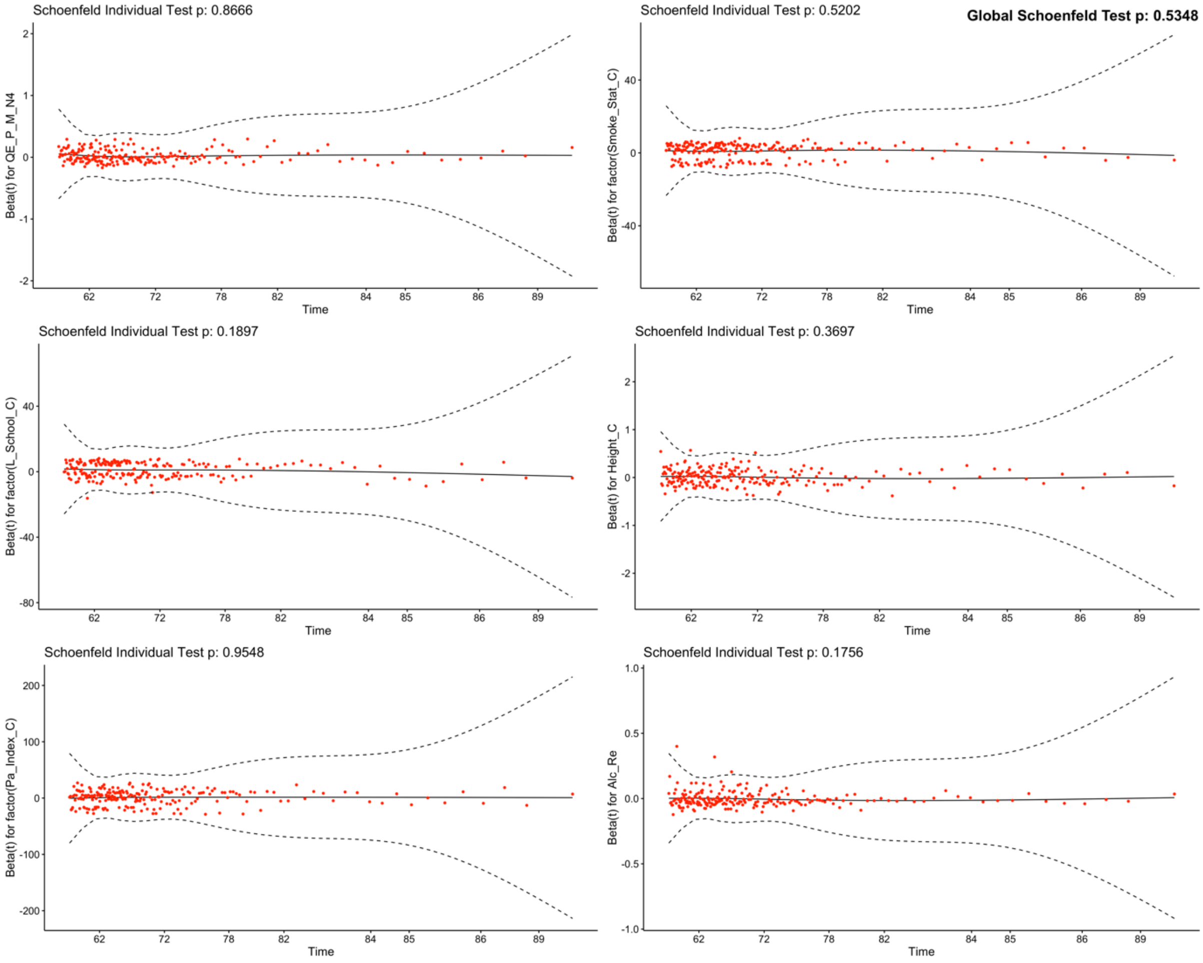

Supplementary Figure 7. Correlation plot for ultra-processed food consumption models.

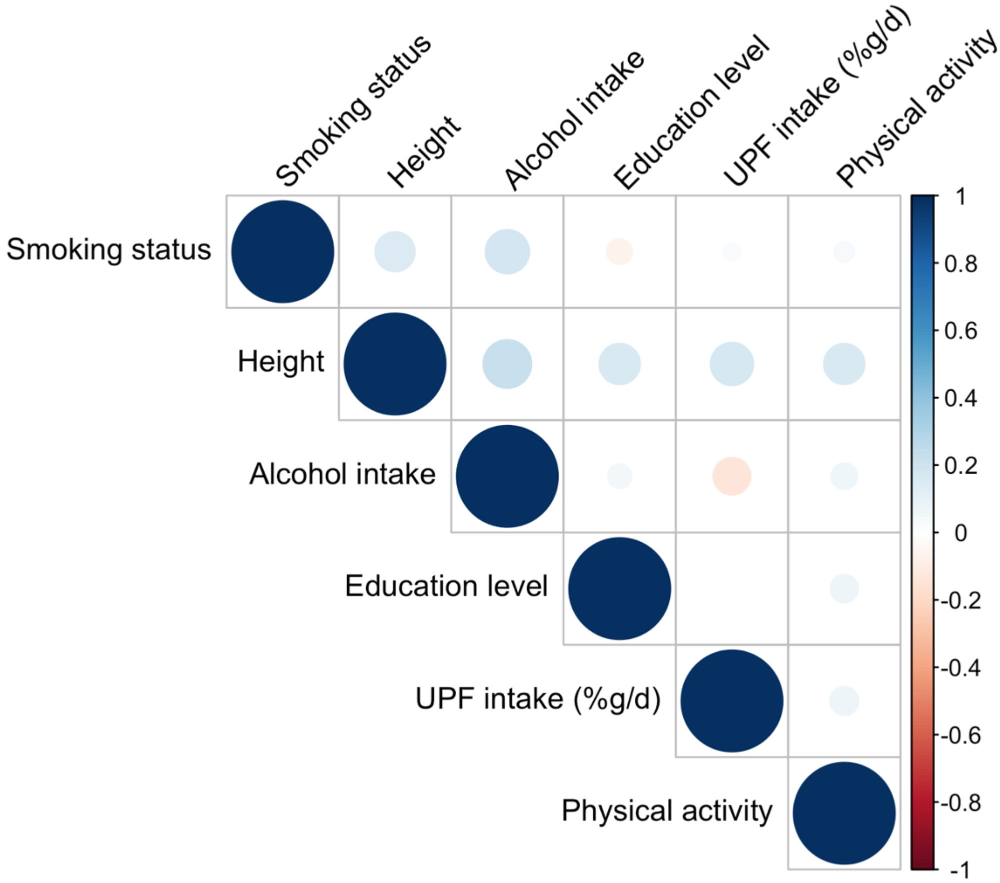

Abbreviations: UPF, ultra-processed food.

Supplementary Figure 8. Non-linearity assessment for the relative intake of ultra-processed foods in grams per day and head and neck cancer risk.

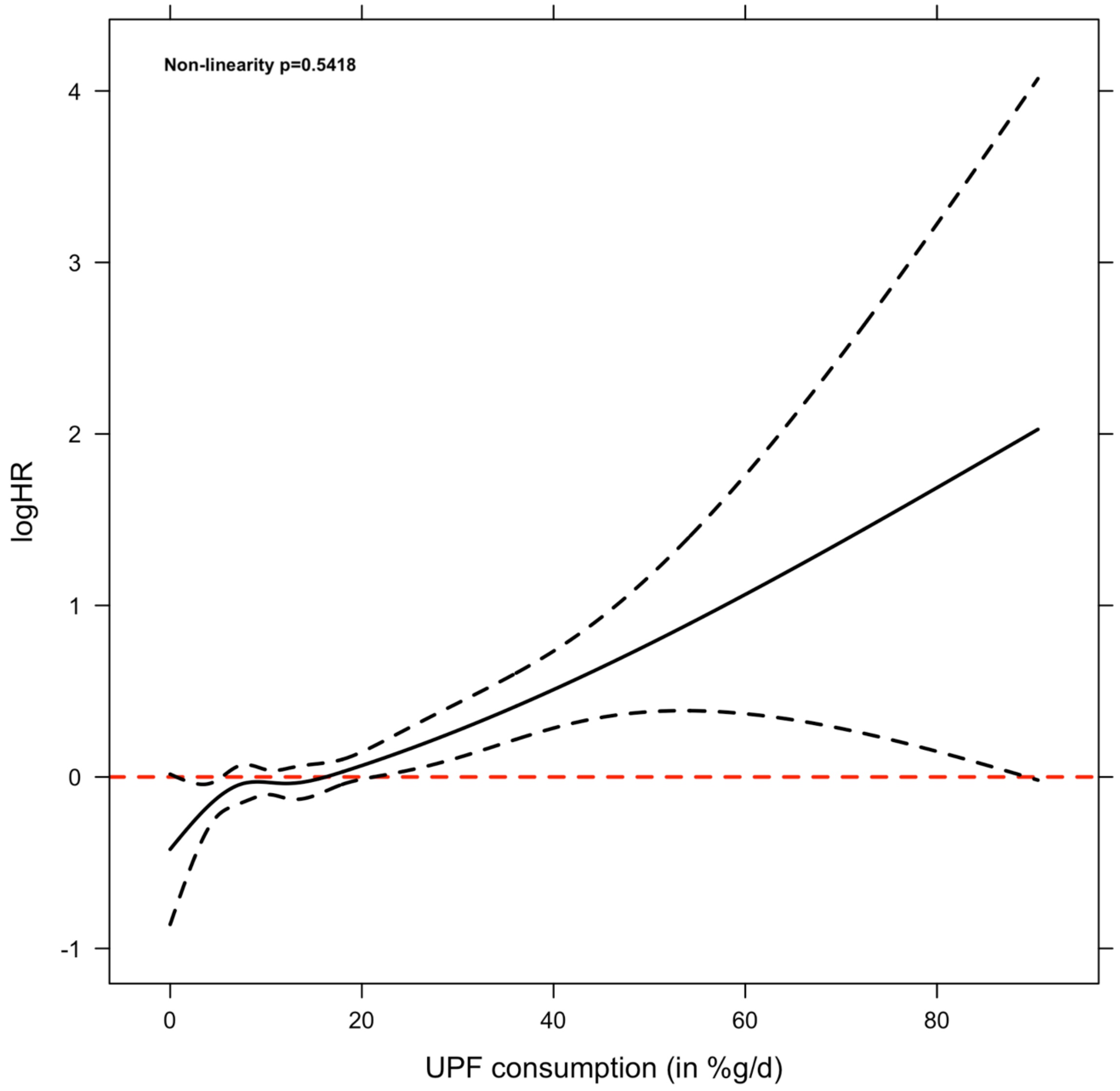

Supplementary Figure 9. Forest plots for associations between the intake of ultra-processed foods (in %g/d, g/d, %kcal/d and kcal/d) and the risk of head and neck cancer and oesophageal adenocarcinoma.

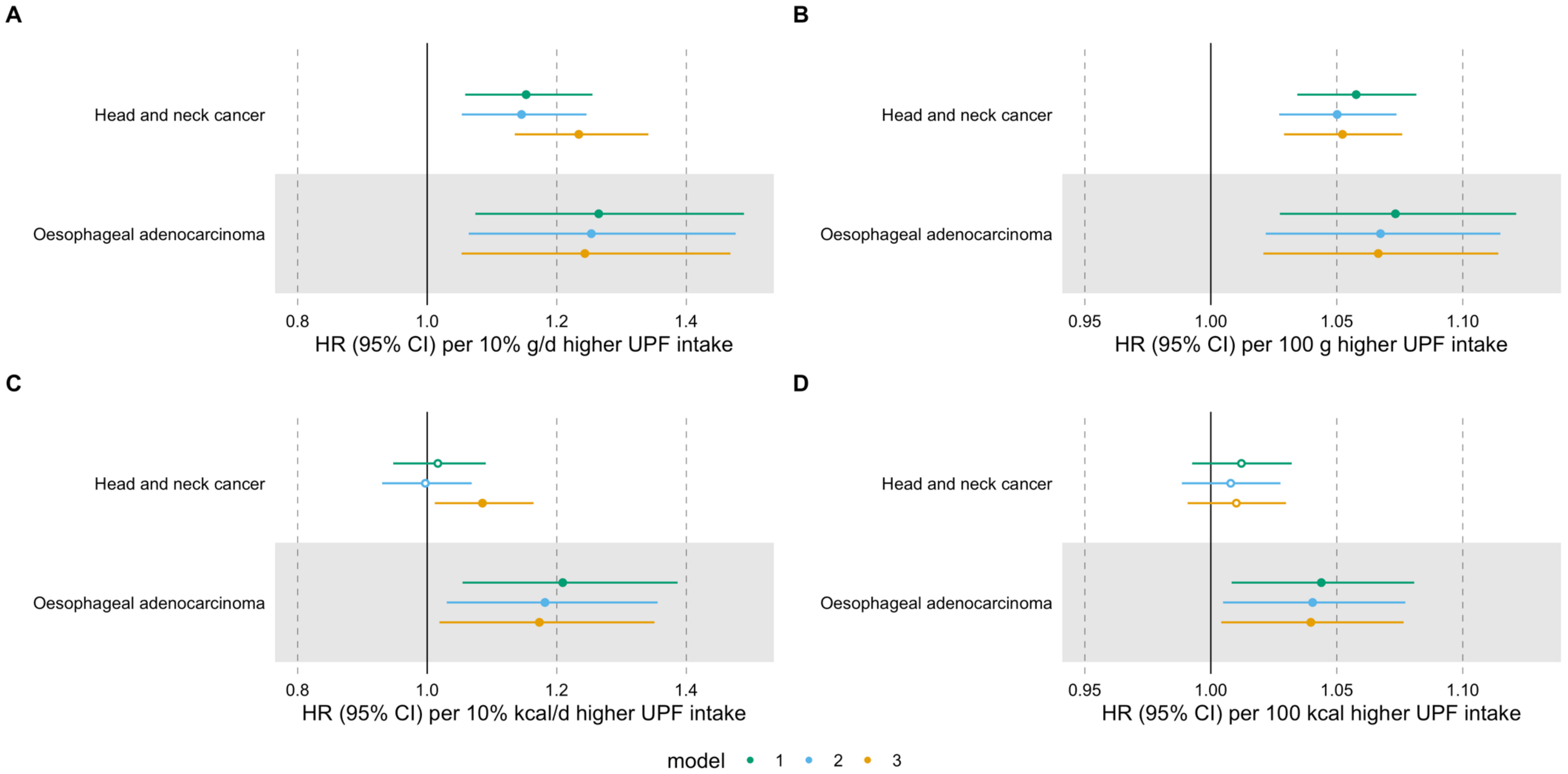

Hazard ratios per **A)** 10% g/d, **B)** 100 g/d, **C)** 10% kcal/d and **D)** 100 kcal/d higher ultra-processed food intake. Time of entry was defined as age at recruitment, while time of exit was defined as age at first cancer diagnosis (excluding non-melanoma skin cancer) or age at last follow-up (i.e., death, emigration, loss to follow-up or end of follow-up), whichever came first. Model 1 was stratified by age at recruitment in 1-year categories, sex, and sub-centre. Model 2 was additionally adjusted for education, physical activity, height, and smoking status. Model 3 was additionally adjusted for alcohol intake. N=450,111 participants, including 910 cases of head and neck cancer and 215 cases of oesophageal adenocarcinoma. Abbreviations: HR, hazard ratio; CI, confidence interval; UPF, ultra-processed foods.

Supplementary Table 1. Food categories contributing to each NOVA group.

	Relative intake (%g/d)	Absolute intake (g/d)
**Total unprocessed/minimally processed foods, mean (SD)**	**71.5 (12.10)**	**1960 (833)**
Water, mean (SD)	10.30 (13.70)	340 (498)
Fruit, mean (SD)	8.95 (7.61)	220 (178)
Milk and plain yoghurt, mean (SD)	9.20 (7.81)	243 (222)
Cereal, grains and flour, mean (SD)	1.65 (2.01)	41.9 (53.40)
Potatoes, mean (SD)	3.32 (2.76)	85.0 (69.40)
Pasta, mean (SD)	1.66 (2.46)	37.7 (49.60)
Beans, lentils and chickpeas, mean (SD)	0.80 (1.20)	19.10 (26.20)
Vegetables, mean (SD)	6.90 (4.75)	175 (117)
Nuts and seeds, mean (SD)	0.08 (0.21)	2.14 (5.36)
Eggs, mean (SD)	0.71 (0.68)	18.00 (17.20)
Poultry, mean (SD)	0.76 (0.87)	18.30 (18.90)
Red meat, mean (SD)	1.81 (1.50)	46.70 (38.20)
Fish, mean (SD)	1.00 (1.39)	22.70 (27.00)
Seafood, mean (SD)	0.127 (0.25)	2.91 (5.57)
Fungi, mean (SD)	0.21 (0.31)	5.73 (8.64)
Coffee/tea, mean (SD)	22.00 (13.70)	632 (481)
Fresh fruit juice and smoothies, mean (SD)	0.37 (1.00)	9.71 (24.70)
Pasteurised fruit juice, mean (SD)	1.56 (2.65)	41.60 (71.60)
Homemade broth, mean (SD)	0.10 (0.27)	2.99 (7.81)
**Total processed ingredients, mean (SD)**	**1.19 (1.04)**	**28.70 (23.50)**
Sugar, mean (SD)	0.39 (0.58)	9.47 (14.10)
Plant-based oil, mean (SD)	0.46 (0.65)	10.00 (13.10)
Animal fats, mean (SD)	0.30 (0.41)	7.75 (10.60)
Other processed culinary ingredients, mean (SD)	0.04 (0.08)	1.03 (2.41)
Salt, mean (SD)	0.02 (0.02)	0.48 (0.84)
**Total processed foods, mean (SD)**	**13.6 (10.00)**	**357 (308)**
Cheese, mean (SD)	1.47 (1.42)	36.60 (33.50)
Salted, smoked and canned meat, mean (SD)	0.33 (0.76)	7.41 (15.40)
Salted, smoked and canned meat with additives, mean (SD)	0.29 (0.44)	7.37 (10.80)
Processed bread, mean (SD)	3.39 (4.01)	81.00 (90.50)

	Relative intake (%g/d)	Absolute intake (g/d)
Preserved vegetables and other plant-based foods, mean (SD)	0.73 (1.24)	18.00 (28.00)
Preserved legumes, mean (SD)	0.18 (0.60)	4.27 (12.50)
Preserved fruit, mean (SD)	0.62 (0.88)	16.60 (24.00)
Salted nuts and nut spreads, mean (SD)	0.07 (0.20)	1.79 (5.00)
Beer and wine, mean (SD)	5.73 (8.10)	165 (276)
Condensed milk and sweetened yoghurt, mean (SD)	0.23 (0.63)	6.29 (18.10)
Breadcrumbs, mean (SD)	0.01 (0.03)	0.31 (0.77)
Artisanal meringue, mean (SD)	0.35 (0.72)	8.73 (19.10)
Artisanal sweet/savoury sauces, mean (SD)	0.17 (0.36)	3.83 (7.82)
**Total ultra-processed foods, mean (SD)**	**13.7 (8.76)**	**364 (264)**
Ultra-processed breads, mean (SD)	1.67 (2.78)	43.60 (67.40)
Pastries, buns and cakes, mean (SD)	0.87 (1.22)	22.10 (29.20)
Biscuits, mean (SD)	0.59 (0.88)	14.20 (18.60)
Breakfast cereals, mean (SD)	0.18 (0.38)	5.03 (10.30)
Ice cream, ice pops and frozen yoghurts, mean (SD)	0.31 (0.55)	7.50 (12.30)
Industrial desserts, mean (SD)	0.07 (0.40)	1.68 (10.90)
Packaged salty snacks, mean (SD)	0.08 (0.22)	2.05 (5.82)
Potato products, mean (SD)	0.32 (0.65)	8.83 (17.80)
Pizza and focaccia dough, mean (SD)	0.27 (0.51)	6.33 (11.00)
Filled pasta, mean (SD)	0.14 (0.39)	3.17 (8.10)
Instant and canned soups, mean (SD)	0.37 (0.82)	9.72 (21.60)
Dairy substitutes, mean (SD)	0.13 (1.33)	3.26 (33.50)
Ultra-processed cheese, mean (SD)	0.13 (0.31)	2.96 (6.86)
Sauces, dressing and gravies (liquid, powder, dehydrated and condensed), mean (SD)	0.37 (0.45)	10.60 (13.50)
Vegetable spreads, mean (SD)	0.01 (0.05)	0.23 (1.45)
Fizzy drinks, mean (SD)	1.95 (4.40)	51.20 (123)
Ultra-processed dairy desserts and drinks, mean (SD)	1.62 (2.42)	43.70 (69.30)
Fruit drinks, iced tea and other sweetened beverages, mean (SD)	1.47 (3.69)	44.00 (123)
Beverages (dry weight), mean (SD)	0.03 (0.15)	0.92 (3.81)
Alcoholic distilled drinks, mean (SD)	0.30 (0.70)	8.42 (21.00)
Artificial sweeteners, mean (SD)	0.01 (0.07)	0.36 (2.18)
Sweet snacks, mean (SD)	0.43 (0.70)	12.00 (19.50)
Ultra-processed meats (beef, pork and fish), mean (SD)	1.46 (1.46)	36.20 (33.50)

	Relative intake (%g/d)	Absolute intake (g/d)
Meat alternatives, mean (SD)	0.03 (0.15)	0.81 (3.85)
Nutrition powders and drinks, mean (SD)	0.00 (0.02)	0.01 (0.53)
Margarine, mean (SD)	0.50 (0.66)	13.10 (16.80)
Ready meals, mean (SD)	0.18 (0.40)	5.68 (12.90)
Alcohol-free versions of alcoholic beverages, mean (SD)	0.09 (0.93)	2.66 (31.20)
Ultra-processed vegetables and legumes, mean (SD)	0.13 (0.46)	3.53 (13.00)
Ultra-processed rice-based dishes, mean (SD)	0.00 (0.01)	0.00 (0.29)

- The contribution of each food category to its corresponding NOVA group can be calculated as follows: mean absolute intake of food category of interest * 100 / mean absolute intake of all foods in corresponding NOVA group.

Supplementary Table 2. Variance inflation factor (VIF) for the assessment of multicollinearity.

	VIF for head and neck cancer	VIF for oesophageal adenocarcinoma
Ultra-processed food consumption in %g/d	1.04	1.04
Smoking status	1.02	1.02
Education level	1.04	1.04
Height	1.03	1.02
Physical activity index	1.01	1.01
Alcohol intake	1.05	1.05

Supplementary Table 3. Associations between the relative intake of ultra-processed foods (in %g/d) and the risk of head and neck cancer and oesophageal adenocarcinoma.

Exposure	Outcome	Model	N events	HR	95% CI	P-value
**Ultra-processed foods**	Head and neck cancer	1	910	1.15	1.06-1.26	0.001
		2	910	1.15	1.05-1.25	0.001
		3	910	1.23	1.14-1.34	<0.001
	Oesophageal adenocarcinoma	1	215	1.26	1.07-1.49	0.005
		2	215	1.25	1.06-1.48	0.007
		3	215	1.24	1.05-1.47	0.01

Hazard ratios per 10% g/d higher ultra-processed food intake. Time of entry was defined as age at recruitment, while time of exit was defined as age at first cancer diagnosis (excluding non-melanoma skin cancer) or age at last follow-up (i.e., death, emigration, loss to follow-up or end of follow-up), whichever came first. Model 1 was stratified by age at recruitment in 1-year categories, sex, and sub-centre. Model 2 was additionally adjusted for education, physical activity, height, and smoking status. Model 3 was additionally adjusted for alcohol intake. N=450,111 participants.

Supplementary Table 4. Associations between the relative intake of ultra-processed foods (in %g/d) and the risk of head and neck cancer subtypes.

Exposure	Outcome	Model	N events	HR	95% CI	P-value	P-value for heterogeneity*
**Ultra-processed foods**	Oral cavity cancer	1	234	1.01	0.84-1.22	0.887	0.107
		2	234	1.01	0.84-1.22	0.899	
		3	234	1.10	0.91-1.32	0.333	
	Oropharynx cancer	1	235	1.15	0.98-1.35	0.090	
		2	235	1.15	0.98-1.34	0.095	
		3	235	1.24	1.05-1.45	0.009	
	Hypopharynx cancer	1	66	1.48	1.16-1.90	0.002	
		2	66	1.43	1.12-1.82	0.004	
		3	66	1.61	1.26-2.06	<0.001	
	Larynx cancer	1	310	1.21	1.05-1.39	0.010	
		2	310	1.20	1.04-1.38	0.011	
		3	310	1.28	1.11-1.47	<0.001	
	Unspecified/ overlapping cancer	1	65	0.96	0.66-1.39	0.819	
		2	65	0.95	0.66-1.36	0.766	
		3	65	0.99	0.69-1.43	0.978	

Hazard ratios per 10% g/d higher ultra-processed food intake. Time of entry was defined as age at recruitment, while time of exit was defined as age at first cancer diagnosis (excluding non-melanoma skin cancer) or age at last follow-up (i.e., death, emigration, loss to follow-up or end of follow-up), whichever came first. Model 1 was stratified by age at recruitment in 1-year categories, sex, and sub-centre. Model 2 was additionally adjusted for education, physical activity, height, and smoking status. Model 3 was additionally adjusted for alcohol intake. N=450,111 participants.
*The P-value for heterogeneity across subtypes was calculated for Model 3 (the fully adjusted model)

Supplementary Table 5. Stratified analyses for the associations between the consumption of ultra-processed foods (in %g/d) and the risk of head and neck cancer and oesophageal adenocarcinoma.

Subgroup variable	Outcome	Subgroup level	N total	N events	HR	95% CI	P-value	P-value for interaction
**Alcohol intake**	Head and neck cancer	No/light alcohol intake	191,805	278	1.21	1.07-1.38	0.003	0.456
		Moderate alcohol intake	188,881	286	1.23	1.05-1.43	0.011	
		Heavy alcohol intake	69,425	346	1.08	0.91-1.27	0.399	
	Oesophageal adenocarcinoma	No/light alcohol intake	191,805	98	1.19	0.94-1.50	0.150	0.179
		Moderate alcohol intake	188,881	67	1.60	1.20-2.15	0.002	
		Heavy alcohol intake	69,425	50	1.00	0.63-1.58	0.998	
**Physical activity index**	Head and neck cancer	Inactive	88,032	212	1.34	1.15-1.56	<0.001	0.484
		Moderately inactive	158,765	297	1.19	1.02-1.39	0.029	
		Moderately active	120,199	203	1.14	0.94-1.38	0.183	
		Active	83,115	198	1.19	0.99-1.44	0.067	
	Oesophageal adenocarcinoma	Inactive	88,032	58	1.26	0.92-1.74	0.152	0.937
		Moderately inactive	158,765	69	1.28	0.94-1.75	0.118	
		Moderately active	120,199	47	1.27	0.89-1.82	0.191	
		Active	83,115	41	1.09	0.74-1.60	0.680	
**Smoking status**	Head and neck cancer	Never	227,717	194	1.06	0.86-1.30	0.570	0.458
		Former	122,680	232	1.23	1.04-1.45	0.017	
		Current	99,714	484	1.30	1.16-1.45	<0.001	
	Oesophageal adenocarcinoma	Never	227,717	57	1.23	0.89-1.72	0.210	0.989
		Former	122,680	94	1.22	0.93-1.60	0.148	
		Current	99,714	64	1.30	0.97-1.73	0.080	
**Sex**	Head and neck cancer	Male	131,425	603	1.34	1.22-1.48	<0.001	0.006
		Female	318,686	307	1.03	0.87-1.21	0.745	
	Oesophageal adenocarcinoma	Male	131,425	169	1.29	1.07-1.54	0.008	0.436
		Female	318,686	46	1.07	0.73-1.58	0.731	
**Education**	Head and neck cancer	Primary school or less	143,488	394	1.29	1.15-1.46	<0.001	0.312
		Secondary or technical/profession al school	197,692	365	1.19	1.04-1.37	0.012	
		Higher education	108,931	151	1.11	0.87-1.42	0.394	
	Oesophageal adenocarcinoma	Primary school or less	143,488	104	1.17	0.92-1.47	0.195	0.834
		Secondary or technical/profession al school	197,692	72	1.26	0.95-1.69	0.114	
		Higher education	108,931	39	1.35	0.86-2.10	0.192	

Hazard ratios per 10% g/d higher ultra-processed food intake. Time of entry was defined as age at recruitment, while time of exit was defined as age at first cancer diagnosis (excluding non-melanoma skin cancer) or age at last follow-up (i.e., death, emigration, loss to follow-up or end of follow-up), whichever came first. Models were stratified by age at recruitment in 1-year categories, sex and sub-centre and additionally adjusted for education, physical activity, height, smoking status and alcohol intake. Models were not adjusted for the stratification variable.

Supplementary Table 6. Identification of possible mediators of the associations between ultra-processed food consumption (in %g/d) and the risk of head and neck cancer and oesophageal adenocarcinoma.

Potential mediators	Association between UPF and M, Odds ratio (95%CI)	UPF-adjusted association between M and head and neck cancer, Hazard ratio (95%CI)	UPF-adjusted association between M and oesophageal adenocarcinoma, Hazard ratio (95%CI)
**Waist-to-hip ratio**	✓ (+)<br>0.41 (0.38 to 0.43)	✓ (+)<br>1.02 (1.01 to 1.03)	✓ (+)<br>1.06 (1.04 to 1.08)
**Body mass index**	✓ (+)<br>0.24 (0.22 to 0.26)	✓ (-)<br>0.98 (0.96 to 0.99)	✓ (+)<br>1.08 (1.04 to 1.11)

Ticks represent evidence of an association. Pluses and minuses represent positive and inverse associations, respectively. Mean change in waist-to-hip ratio and body mass index per 10% g/d higher ultra-processed food intake. Hazard ratios per 0.01 higher waist-to-hip ratio and per 1 kg/m^2 higher body mass index. Cox models for the mediator-outcome associations were stratified by age at recruitment in 1-year categories, sex, and sub-centre and additionally adjusted for education, physical activity, height, smoking status, and alcohol intake. Linear models for the exposure-mediator associations were adjusted for the same confounders. Waist-to-hip ratio models included 336,858 participants, of which 828 and 195 had head and neck cancer and oesophageal adenocarcinoma, respectively. Body mass index models included 365,549 participants, of which 864 and 212 had head and neck cancer and oesophageal adenocarcinoma, respectively. Abbreviations: NA, not applicable as there is no evidence of an association between the exposure and the outcome after accounting for all confounders; UPF, ultra-processed foods; M, mediator; CI, confidence interval.

Supplementary Table 7. Associations between the relative intake of ultra-processed foods (in %g/d) and the risk of head and neck cancer and oesophageal adenocarcinoma, accounting for total water intake (including water in foods).

Exposure	Outcome	Model	N events	HR	95% CI	P-value
**Ultra-processed foods**	Head and neck cancer	1	910	1.19	1.10-1.30	<0.001
		2	910	1.17	1.08-1.28	<0.001
		3	910	1.24	1.14-1.34	<0.001
	Oesophageal adenocarcinoma	1	215	1.29	1.10-1.52	0.002
		2	215	1.27	1.08-1.50	0.003
		3	215	1.26	1.07-1.48	0.006

Hazard ratios per 10% g/d higher ultra-processed food intake. Time of entry was defined as age at recruitment, while time of exit was defined as age at first cancer diagnosis (excluding non-melanoma skin cancer) or age at last follow-up (i.e., death, emigration, loss to follow-up or end of follow-up), whichever came first. Model 1 was stratified by age at recruitment in 1-year categories, sex, and sub-centre, and adjusted for water intake (including water in foods). Model 2 was additionally adjusted for education, physical activity, height, and smoking status. Model 3 was additionally adjusted for alcohol intake. N=450,111 participants.

Supplementary Table 8. Associations between the relative intake of ultra-processed foods (in %g/d) and the risk of head and neck cancer and oesophageal adenocarcinoma, accounting for energy intake.

Exposure	Outcome	Model	N events	HR	95% CI	P-value
**Ultra-processed foods**	Head and neck cancer	1	910	1.14	1.05-1.25	0.003
		2	910	1.13	1.04-1.24	0.004
		3	910	1.27	1.17-1.39	<0.001
	Oesophageal adenocarcinoma	1	215	1.25	1.06-1.48	0.009
		2	215	1.24	1.04-1.47	0.014
		3	215	1.22	1.02-1.45	0.027

Hazard ratios per 10% g/d higher ultra-processed food intake. Time of entry was defined as age at recruitment, while time of exit was defined as age at first cancer diagnosis (excluding non-melanoma skin cancer) or age at last follow-up (i.e., death, emigration, loss to follow-up or end of follow-up), whichever came first. Model 1 was stratified by age at recruitment in 1-year categories, sex, and sub-centre, and adjusted for energy intake. Model 2 was additionally adjusted for education, physical activity, height, and smoking status. Model 3 was additionally adjusted for alcohol intake. N=450,111 participants.

Supplementary Table 9. Associations between the relative intake of ultra-processed foods (in %g/d) and the risk of head and neck cancer and oesophageal adenocarcinoma, excluding participants censored in the first two years of follow-up.

Exposure	Outcome	Model	N events	HR	95% CI	P-value
**Ultra-processed foods**	Head and neck cancer	1	818	1.14	1.04-1.24	0.006
		2	818	1.13	1.03-1.24	0.008
		3	818	1.21	1.11-1.33	<0.001
	Oesophageal adenocarcinoma	1	196	1.26	1.06-1.49	0.009
		2	196	1.25	1.05-1.48	0.012
		3	196	1.23	1.03-1.46	0.020

Hazard ratios per 10% g/d higher ultra-processed food intake. Time of entry was defined as age at recruitment, while time of exit was defined as age at first cancer diagnosis (excluding non-melanoma skin cancer) or age at last follow-up (i.e., death, emigration, loss to follow-up or end of follow-up), whichever came first. Model 1 was stratified by age at recruitment in 1-year categories, sex, and sub-centre. Model 2 was additionally adjusted for education, physical activity, height, and smoking status. Model 3 was additionally adjusted for alcohol intake. N=442,536 participants, since 7575 participants were excluded from the analysis because they were censored during the first two years of follow-up.

Supplementary Table 10. Complete case analysis for the associations between the relative intake of ultra-processed foods (in %g/d) and the risk of head and neck cancer and oesophageal adenocarcinoma.

Exposure	Outcome	Model	N events	HR	95% CI	P-value
**Ultra-processed foods**	Head and neck cancer	1	851	1.15	1.06-1.26	0.002
		2	851	1.15	1.05-1.25	0.002
		3	851	1.24	1.14-1.35	<0.001
	Oesophageal adenocarcinoma	1	191	1.26	1.06-1.50	0.009
		2	191	1.25	1.05-1.49	0.013
		3	191	1.24	1.04-1.48	0.016

Hazard ratios per 10% g/d higher ultra-processed food intake. Centre-, age- and sex-specific imputed height was used as a covariate, as this is standard practice when dealing with anthropometric variables as confounders in EPIC. Time of entry was defined as age at recruitment, while time of exit was defined as age at first cancer diagnosis (excluding non-melanoma skin cancer) or age at last follow-up (i.e., death, emigration, loss to follow-up or end of follow-up), whichever came first. Model 1 was stratified by age at recruitment in 1-year categories, sex, and sub-centre. Model 2 was additionally adjusted for education, physical activity, height, and smoking status. Model 3 was additionally adjusted for alcohol intake. N= 419,590.

Supplementary Table 11. Multivariate imputation by chained equations analyses for the associations between the relative intake of ultra-processed foods (in %g/d) and the risk of head and neck cancer and oesophageal adenocarcinoma.

**Exposure**	**Outcome**	**Model**	**N events**	**HR**	**95% CI**	**P-value**
**Ultra-processed foods**	Head and neck cancer	1	910	1.15	1.06-1.26	0.001
		2	910	1.15	1.06-1.25	0.001
		3	910	1.24	1.14-1.34	<0.001
	Oesophageal adenocarcinoma	1	215	1.26	1.07-1.49	0.005
		2	215	1.25	1.07-1.48	0.007
		3	215	1.24	1.05-1.47	0.011

Hazard ratios per 10% g/d higher ultra-processed food intake. Multivariate imputation by chained equations (MICE) was used to impute physical activity, education level and smoking status five times using predictive mean matching. Models were fit using the MICE imputed data sets and then pooled to obtain single estimates and standard errors for each model. Centre-, age- and sex-specific imputed height was used as a covariate, as this is standard practice when dealing with anthropometric variables as confounders in EPIC. Time of entry was defined as age at recruitment, while time of exit was defined as age at first cancer diagnosis (excluding non-melanoma skin cancer) or age at last follow-up (i.e., death, emigration, loss to follow-up or end of follow-up), whichever came first. Model 1 was stratified by age at recruitment in 1-year categories, sex, and sub-centre. Model 2 was additionally adjusted for education, physical activity, height, and smoking status. Model 3 was additionally adjusted for alcohol intake. N= 450,111.

Supplementary Table 12. Associations between the absolute (in g/d and kcal/d) and relative intake (in %kcal/d) of ultra-processed foods and the risk of head and neck cancer and oesophageal adenocarcinoma.

Exposure	Outcome	Model	N events	HR	95% CI	P-value
**Ultra-processed foods in g/d** [a]	Head and neck cancer	1	910	1.06	1.03-1.08	<0.001
		2	910	1.05	1.03-1.07	<0.001
		3	910	1.05	1.03-1.08	<0.001
	Oesophageal adenocarcinoma	1	215	1.07	1.03-1.12	0.002
		2	215	1.07	1.02-1.11	0.003
		3	215	1.07	1.02-1.11	0.004
**Ultra-processed foods in %kcal/d** [b]	Head and neck cancer	1	910	1.02	0.95-1.09	0.649
		2	910	1.00	0.93-1.07	0.936
		3	910	1.09	1.01-1.16	0.022
	Oesophageal adenocarcinoma	1	215	1.21	1.05-1.39	0.007
		2	215	1.18	1.03-1.36	0.017
		3	215	1.17	1.02-1.35	0.026
**Ultra-processed foods in kcal/d** [c]	Head and neck cancer	1	910	1.01	0.99-1.03	0.225
		2	910	1.01	0.99-1.03	0.427
		3	910	1.01	0.99-1.03	0.307
	Oesophageal adenocarcinoma	1	215	1.04	1.01-1.08	0.015
		2	215	1.04	1.00-1.08	0.026
		3	215	1.04	1.00-1.08	0.028

Hazard ratios per [a]100 g/d, [b]10% kcal/d and [c]100 kcal/d higher ultra-processed food intake. Time of entry was defined as age at recruitment, while time of exit was defined as age at first cancer diagnosis (excluding non-melanoma skin cancer) or age at last follow-up (i.e., death, emigration, loss to follow-up or end of follow-up), whichever came first. Model 1 was stratified by age at recruitment in 1-year categories, sex, and sub-centre. Model 2 was additionally adjusted for education, physical activity, height, and smoking status. Model 3 was additionally adjusted for alcohol intake. N=450,111 participants.

Supplementary Table 13. Negative control outcome analysis for the association between the relative intake of ultra-processed foods (in %g/d) and the risk of accidental deaths.

Exposure	Outcome	Model	N events	HR	95% CI	P-value
**Ultra-processed foods**	Accidental death	1	871	1.11	1.01-1.22	0.029
		2	871	1.10	1.00-1.21	0.041
		3	871	1.12	1.02-1.23	0.020

Hazard ratios per 10% g/d higher ultra-processed food intake. Time of entry was defined as age at recruitment, while time of exit was defined as age at death, emigration, loss to follow-up or end of follow-up, whichever came first. Model 1 was stratified by age at recruitment in 1-year categories, sex, and sub-centre. Model 2 was additionally adjusted for education, physical activity, height, and smoking status. Model 3 was additionally adjusted for alcohol intake. N=450,111 participants.
